# Identification of *TYROBP* and *C1QB* as Two Novel Key Genes With Prognostic Value in Gastric Cancer by Network Analysis

**DOI:** 10.3389/fonc.2020.01765

**Published:** 2020-09-11

**Authors:** Junjie Jiang, Yongfeng Ding, Mengjie Wu, Xiadong Lyu, Haifeng Wang, Yanyan Chen, Haiyong Wang, Lisong Teng

**Affiliations:** ^1^Department of Surgical Oncology, The First Affiliated Hospital, Zhejiang University School of Medicine, Hangzhou, China; ^2^Department of Medical Oncology, The First Affiliated Hospital, Zhejiang University School of Medicine, Hangzhou, China; ^3^Department of Hematology & Oncology, The People's Hospital of Beilun District, Beilun Branch Hospital of the First Affiliated Hospital of Medical School of Zhejiang University, Ningbo, China

**Keywords:** gastric cancer, protein-protein interaction, weighted gene co-expression network analysis, biomarker, prognosis

## Abstract

**Background:** Gastric cancer (GC) is the fifth most frequently diagnosed malignancy, and the third leading cause of tumor-related mortalities worldwide. Due to a high heterogeneity in GC, its treatment and prognosis are challenging, necessitating urgent identification of novel prognostic predictors for GC patients.

**Methods:** We downloaded RNA sequence data, from the Cancer Genome Atlas and microarray data from Gene Expression Omnibus database, then identified common differentially-expressed genes (DEGs) between GC and normal gastric tissues across four datasets. We then used a combination of protein-protein interaction (PPI) network and weighted gene co-expression network analysis (WGCNA) to identify key genes with prognostic value in GC. Thereafter, we used quantitative real time polymerase chain reaction (qRT-PCR) to validate expression of the identified key genes in the Zhejiang University (ZJU) cohort. Finally, we evaluated the relationships between gene expression and immune factors, including immune cells and biomarkers of immunotherapy.

**Results:** Among 426 common DEGs screened, 333 and 93 were upregulated and downregulated, respectively. PPI network and WGCNA successfully identified the top 30 hub genes, among which *PTPRC, TYROBP, CCR1, CYBB, LCP2*, and *C1QB* were common. Furthermore, *TYROBP* and *C1QB* were negatively associated with prognosis of GC patients, implying that they were key GC predictors. Interestingly, *TYROBP* and *C1QB* were positively correlated with predictive biomarkers for GC immunotherapy, including PD-L1 expression, CD8^+^ T cells infiltration, and EBV status.

**Conclusions:** *TYROBP* and *C1QB* were identified as two novel key genes with prognostic value in GC by network analysis.

## Introduction

Gastric cancer (GC) is a major human health burden. According to the *GLOBOCAN*, in 2018 alone, there were over 1,000,000 new GC cases and an estimated 783,000 GC-related fatalities, making it the fifth prevalent cancer and the third leading cause of tumor mortality (1). Due to its high recurrence after surgery (2) and low sensitivity to chemotherapy (3), the overall 5-year survival rate of GC patients remains low. Therefore, it is urgent and crucial to identify novel prognostic biomarkers for GC patients.

With the rapid development and extensive application of high-throughput technology, vast amounts of gene expression profiles have been produced and utilized to identify differentially expressed genes (DEGs) by comparing tumor cells with the adjacent mucosa (4). However, the previous conventional studies have focused more on the individual DEGs while ignoring the complex network with a high degree of interconnection between the DEGs. Protein–protein interaction (PPI) networks and weighted gene co-expression network analysis (WGCNA) based on the microarray and RNA sequencing data have been shown to constitute powerful systematical biology strategies for mining the functional gene modules and identifying hub genes as candidate biomarkers, as well as therapeutic targets (5, 6). Over the past years, PPI and WGCNA have been extensively applied to screen out hub genes in multiple cancers. For instance, Chen et al. identified and validated that *VCAN* is associated with the progression and prognosis of pancreatic cancer by constructing a PPI network (7). Similarly, Yin et al. identified three novel blood-based diagnostic biomarkers for human hepatocellular carcinoma by WGCNA (8).

Herein, we constructed PPI and WGCNA networks based on the common DEGs from the TCGA-STAD (9) and 3 Gene Expression Omnibus (GEO) datasets [GSE65801 (10), GSE54129, and GSE118916 (11)]. Hub modules and hub genes were screened from the networks. An integrated bioinformatics analysis was performed to evaluate the function, pathway, and interrelation of the hub modules and the hub genes. We identified the key genes via survival analysis from the common hub genes derived from the PPI and WGCNA network, then validated them in the Oncomine database, ZJU cohort, and GSE15459 dataset (12). An immune analysis was performed to investigate the association between the key genes and the immune factors using the TCGA-STAD and GSE51575 dataset (13).

## Materials and Methods

### Study Design

**Figure 1:**
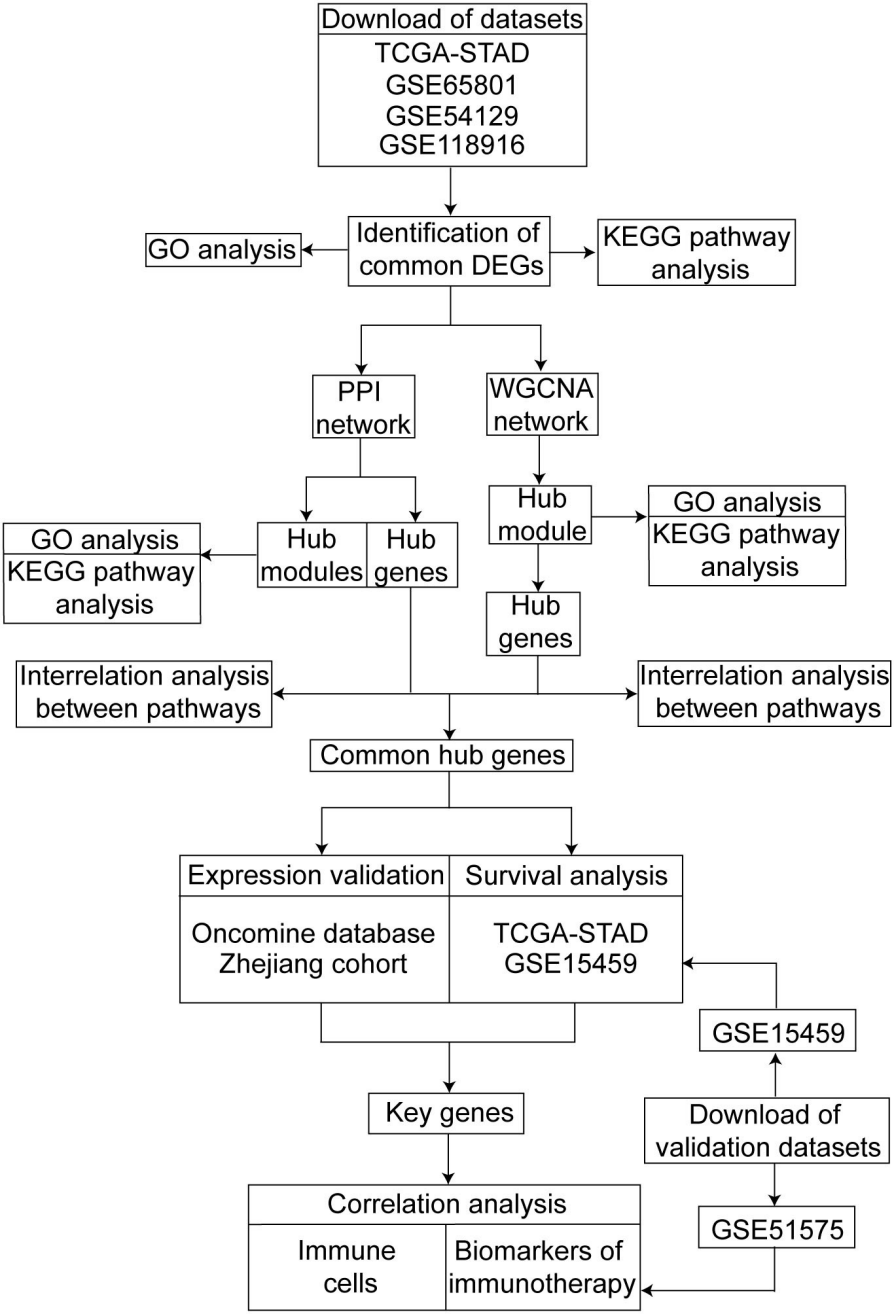
Workflow of our study for identifying key genes with prognostic value in gastric cancer, including data preparing, processing and analysis.

The design of this study is shown as a workflow (Figure 1). We screened the differentially expressed genes (DEGs) between the GC and normal or adjacent mucosa tissue from the four cohort profile datasets, i.e., TCGA-STAD (9), GSE65801 (10), GSE54129, and GSE118916 (11). The construction of the protein–protein interaction (PPI) network and weighted gene co-expression network was based on the DEGs, and we identified the common hub genes from the networks. The expression of the common hub genes was validated in the Oncomine database and ZJU cohort. We performed survival analyses of the common hub genes using the TCGA-STAD dataset. Immune analyses were performed to evaluate the correlation between the key genes and the tumor microenvironment using the TCGA-STAD and GSE51575 datasets (13).

### Data Collection

We downloaded the RNA sequencing data and clinical datasets of GC patients from the TCGA repository of the National Cancer Institute (https://cancergenome.nih.gov/). The TCGA-STAD datasets constituted 375 tumor and 32 normal samples. Microarray data of GSE65801, GSE54129, GSE118916, GSE15459, and GSE51575 datasets were retrieved from the Gene Expression Omnibus (GEO) database (https://www.ncbi.nlm.nih.gov/geo). The GSE65801 microarray data was downloaded from the GPL14550 Platform (Agilent-028004 SurePrint G3 Human GE 8x60K Microarray, Probe Name version, Agilent Technologies) and included 32 gastric cancer tissues and 32 paired noncancerous tissues (Submission date: Feb 10, 2015) (10). The microarray data of GSE54129 and GSE15459 was downloaded from the GPL570 Platform ([HG-U133_Plus_2] Affymetrix Human Genome U133 Plus 2.0 Array), with the former constituting 111 gastric cancer tissues and 21 normal gastric tissues (Submission date: Jan 16, 2014) while the latter included 200 primary gastric cancer tissues (Submission date: Mar 30, 2009) (12). The GSE118916 microarray data was downloaded from the GPL15207 Platform ([PrimeView] Affymetrix Human Gene Expression Array) and included 15 gastric cancer tissues and 15 paired adjacent mucosa tissues (Submission date: Aug 22, 2018) (11). The GSE51575 microarray data was downloaded from the GPL13607 Platform (Agilent-028004 SurePrint G3 Human GE 8x60K Microarray, Feature Number version) and included 26 adjacent mucosa tissues, 14 EBV-positive gastric cancer tissues, and 12 EBV-negative gastric cancer tissues (Submission date: Oct 23, 2013) (13). The GSE51575 dataset was derived from a primary study (13) and contained some essential information for our research, including gene expression of immune checkpoints, and EBV infection status. The acquisition and application methods of all the data were according to the guidelines and policies of the GEO and TCGA databases.

### Data Preprocessing and Common DEGs Identification

The retrieved gene expression data from the GEO database was preprocessed, including background correction and normalization in the R version 3.6.1 software. We utilized the Bioconductor Annotation Data software package to transform the microarray data probes to gene symbols. When several probes were matched to the same gene symbol, the median value was set as the final expression value of the gene. The “limma” and “edgeR” R packages were utilized to identify the DEGs between the GC tissues and normal or adjacent mucosa tissues in the GEO and TCGA datasets, respectively (14, 15). Genes with adjusted *P* < 0.05 and |Fold change (FC)| > 1.5 were selected as the DEGs. Common DEGs were defined as the overlap of the DEGs from the TCGA-STAD, GSE65801, GSE54129, and GSE118916. The Venn diagram was generated online (http://bioinformatics.~psb.ugent.be/webtools/Venn/).

### Functional Annotation, Pathway Enrichment, and Interrelation Analysis

We analyzed the functional annotation and pathway enrichment using the Database for Annotation, Visualization and Integrated Discovery (DAVID) web portal (https://david.ncifcrf.gov/) (16, 17). After uploading the list of common DEGs, we obtained the Gene Ontology (GO) enrichment results of the biological process (BP), cellular component (CC), molecular function (MF), and the Kyoto Encyclopedia of Genes and Genomes (KEGG) pathway. Interrelation analysis between pathways and hub genes was performed using the ClueGo (Version 2.5.4), a plug-in of Cytoscape software (18). *P* < 0.05 was set as the cut-off criteria.

### PPI Network Construction and MCODE Analysis

First, we utilized the STRING database (http://string-db.org) to construct the PPI network of DEGs and interactions, with a combined score > 0.4 considered statistically significant (6). After that, we used the Cytoscape software (Version 3.7.2) to visualize the PPI network (19). Subsequently, the Molecular Complex Detection (MCODE, version 1.5.1) plug-in tool of Cytoscape was used to screen and visualize the hub modules in the PPI network with the MCODE score = 5, degree = 2, Node score cut-off = 0.2, K-score = 2, and Max. Depth = 100 (20). The functional annotation for the genes in the modules was performed using the DAVID.

### WGCNA Network Construction

We utilized the WGCNA to analyze the co-expressed gene module and identify the hub module correlated to the clinical traits (5). In this study, we selected the common DEGs for the WGCNA network construction using the “WGCNA” R package. Sample clustering of the common DEGs was applied to filter the outlier sample with a height cut-off value of 20,000. A power of β = 4 and minimum module size = 30 were set as per the standard scale-free networks. The adjacencies between all the filtered genes were conducted and converted into a topological overlap matrix (TOM) and the corresponding dissimilarity (1-TOM). The hierarchical clustering function was used to classify the genes with a high absolute correlation into modules based on the TOM-based dissimilarity for the gene dendrogram. The dissimilarity of the module eigengenes was calculated to merge similar modules with a height cut-off value of 0.25. Module eigengene (ME), defined as the first principal component of a given module, was regarded as the representative of the module. The correlation between the ME and the clinical traits, including age, gender, grade, and the stage, was one of the factors for identifying the hub module. Gene significance (GS) was defined as the log10 transformation of *P*-value in the linear regression between the gene expression and the clinical traits. The module membership (MM) was identified as the correlation between the gene expression and the ME. The hub module was identified by the highest correlation between the ME and the clinical traits, as well as the most significant correlation between the MM and the GS. Subsequently, the hub module was visualized using the Cytoscape software. The functional annotation and pathway enrichment for the genes in the hub module were conducted using DAVID.

### Common Hub Genes Identification and Validation

The highly interconnected hub genes with the other nodes in a module were regarded as functionally significant genes. Herein, the hub modules were identified using the PPI network and the WGCNA network. The hub genes in the hub modules were screened using the cytoHubba (Version 0.1) tool, a plug-in of the Cytoscape software (21). The hub genes that ranked the top 30 in the hub modules were selected as the candidates using the Degree method, and the interrelation analysis was performed, as described previously. The common hub genes defined as the overlap of the hub genes from the PPI network and the WGCNA network were identified for further analysis and validation. The expression of the common hub genes was validated using the Oncomine database.

### Tissue Samples and Total RNA Isolation

We obtained 10 pairs of the GC tissues and adjacent mucosa tissues from GC patients who underwent surgery at the First Affiliated Hospital of Zhejiang University (ZJU cohort), excluding those who had been exposed to pre-operative chemotherapy or radiotherapy. The Institutional Review Board of the First Affiliated Hospital of Zhejiang University approved the protocol of this study. All the GC patients signed informed consent. The total RNA from each of the 10 GC tissues and 10 paired adjacent mucosa tissues was isolated using a RNeasy Mini Kit (Cat.no.74106, Qiagen, Germany) and quantified using a NanoDrop One (Cat. ND-ONE-W, ThermoFisher Scientific, USA).

### Quantitative Real-Time Polymerase Chain Reaction (qRT-PCR)

Briefly, we performed reverse transcription to synthesize the first-strand cDNA using 1 μg total RNA isolated from the GC tissues and paired normal mucosa tissue samples using the PrimeScript™ RT Master Mix (Perfect Real Time) (Cat. #RR036A, TaKaRa, Japan). After that, qRT-PCR was performed using the TBGreen®Premix Ex Taq™II (Tli RNase H Plus) (Cat. #RR820A, TaKaRa, Japan). We utilized the Glyceraldehyde 3-phosphate dehydrogenase (GAPDH) gene as an internal control.

### Survival Analysis and Key Genes Identification

We assessed the prognostic value of the common hub genes by evaluating the association of the gene expression and overall survival of patients in the TCGA-STAD dataset. The key genes with clinical significance were identified through expression validation and survival analysis of the common hub genes. Finally, the transcriptional expression of the key genes was validated in the ZJU cohort.

### Immune Analysis of Key Genes

As an interactive web platform, Tumor Immune Estimation Resource (TIMER) (https://cistrome.shinyapps.io/timer/) is utilized to estimate tumor immune infiltration across diverse cancer types (22). In this study, we analyzed the correlation of the expression of key genes in GC with immune infiltration (CD8^+^ T Cells, CD4^+^ T Cells, and Macrophages) using the “Gene” module and explored the correlation between immune infiltration and survival using the “Survival” module in TIMER. CIBERSORT is a deconvolution algorithm used to estimate the immune cell type proportions with a signature matrix of 547 genes by support vector regression (23). The output includes a *P*-value for the deconvolution of each sample using the Monte Carlo sampling after running with 1,000 permutations. The CIBERSORT *P*-value reflects the statistical significance of the results, and a threshold < 0.05 is recommended. We uploaded the gene expression profile constituting 375 tumor samples in the TCGA-STAD to the CIBERSORT web portal (https://cibersort.stanford.edu/). Consequently, 240 samples with CIBERSORT *P* < 0.05 were included in calculating the Spearman's correlation between the key genes and 22 types of the infiltrating immune cells. Besides, we evaluated the correlation between the key genes and the immune factors, including cytolytic activity molecules (GZMA and PRF1) and the immune checkpoints (CD274, PDCD1, PDCD1LG2, VTCN, and LAG3) of 375 samples in the TCGA-STAD dataset. The correlation between the key genes and the immune checkpoints was validated using the GSE51575 data. Information about the TCGA-STAD subtypes, including CIN, EBV, MSI, and GS, was mined from the cBioportal database (24), an open-access resource providing data from the TCGA project (https://www.cbioportal.org/). Epstein Barr virus (EBV) associated with gastric cancer was classified as one of the four molecular subtypes in 2014 (9). EBV positive status additionally was one of the biomarkers for immunotherapy. We then evaluated the association between the key genes and the EBV status in the TCGA-STAD and GSE51575 datasets. The correlation between the key genes and immune factors in the TCGA-STAD dataset was visualized using the MeV (MultiExperiment Viewer, version 4.9.0.) software (25).

### Correlation Analysis of Key Genes

The correlation analysis of the key genes was performed using the Gene Expression Profiling Interactive Analysis (GEPIA) (26), a web server for analyzing gene expression from the TCGA and GTEx samples (http://gepia2.cancer-pku.cn/).

### Statistical Analysis

Statistical analyses were performed using the SPSS 21.0 software. We used the Kaplan-Meier survival to analyze the association between gene expression and the overall survival. The log-rank test was used to determine significant differences in the survival curves stratified by the gene expression level. We calculated the median overall survival time, and the 95% confidence interval where relevant. The correlations of the gene expression with immune cells and immune factors were evaluated using the Spearman's correlation and statistical significance. The continuous variables in the two groups and multi-subgroups were compared using the Student's *t*-test and ANOVA, respectively. A *P* < 0.05 was considered statistically significant.

## Results

### Identification, Functional Annotation, and Pathway Analysis of Common DEGs

**Figure 2:**
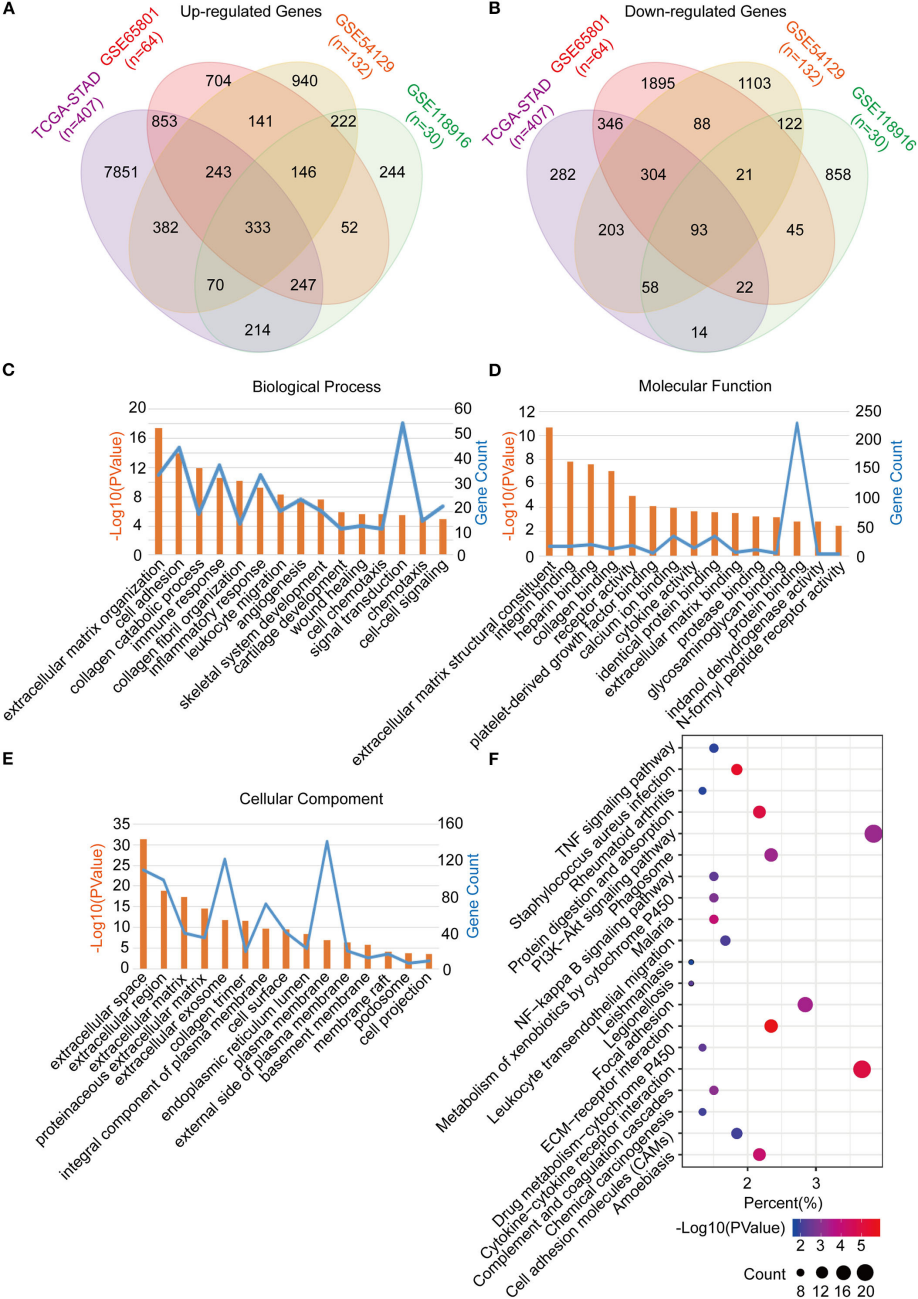
Identification, functional annotation and pathway analysis of the common DEGs in four cohorts (TCGA-STAD, GSE65801, GSE54129, and GSE118916). **(A)** Venn diagram of the up-regulated genes in the four cohorts. **(B)** Venn diagram of the down-regulated genes in the four cohorts. **(C)** Biological processes of the common DEGs. **(D)** Cellular components of the common DEGs. **(E)** Molecular functions of the common DEGs. **(F)** KEGG pathway analysis of the common DEGs.

The common DEGs between the GC tumor and normal or adjacent mucosa tissues were screened from the GEO datasets (GSE65801, GSE54129, and GSE118916), and the TCGA-STAD dataset. Consequently, 426 common DEGs were identified, of which 333 were upregulated, and 93 were downregulated (Figures 2A,B; Table S1). After that, we performed Go analyses by uploading the identified 426 common DEGs to the DAVID web portal. The DEGs were enriched as per the four subontologies: BP, CC, MF, and the KEGG pathway. For BP (Figure 2C), the DEGs were primarily enriched in signal transduction, cell adhesion, and immune response. The other biological processes were associated with the tumor microenvironment constituted the extracellular matrix organization, collagen catabolic process, collagen fibril organization, inflammatory response, leukocyte migration, angiogenesis, chemotaxis, and cell-cell signaling. For MF (Figure 2D), enrichment of the DEGs was primarily in protein binding and calcium ion binding. The other molecular functions were mainly enriched in receptor activity included integrin binding, heparin binding, collagen binding, platelet-derived growth factor binding, cytokine activity, identical protein binding, extracellular matrix binding, protease binding, glycosaminoglycan binding, indanol dehydrogenase activity, and the N-formyl peptide receptor activity. For CC (Figure 2E), the DEGs were mainly involved in the plasma membrane, extracellular space, and exosome. The other cellular components included extracellular region, extracellular matrix, proteinaceous extracellular matrix, collagen trimer, integral component of the plasma membrane, cell surface, endoplasmic reticulum lumen, plasma membrane, external side of the plasma membrane, basement membrane, membrane, membrane raft, podosome, and the cell projection. For the KEGG pathway (Figure 2F), the DEGs were mainly enriched in the PI3K-Akt signaling pathway, cytokine-cytokine receptor interaction, and focal adhesion. The other pathways were mainly enriched in immune signaling, including the TNF signaling pathway, staphylococcus aureus infection, rheumatoid arthritis, leukocyte transendothelial migration, and chemical carcinogenesis.

### Identification, Enrichment, and Interrelation Analysis of Hub Modules and Hub Genes in the PPI Network

**Figure 3:**
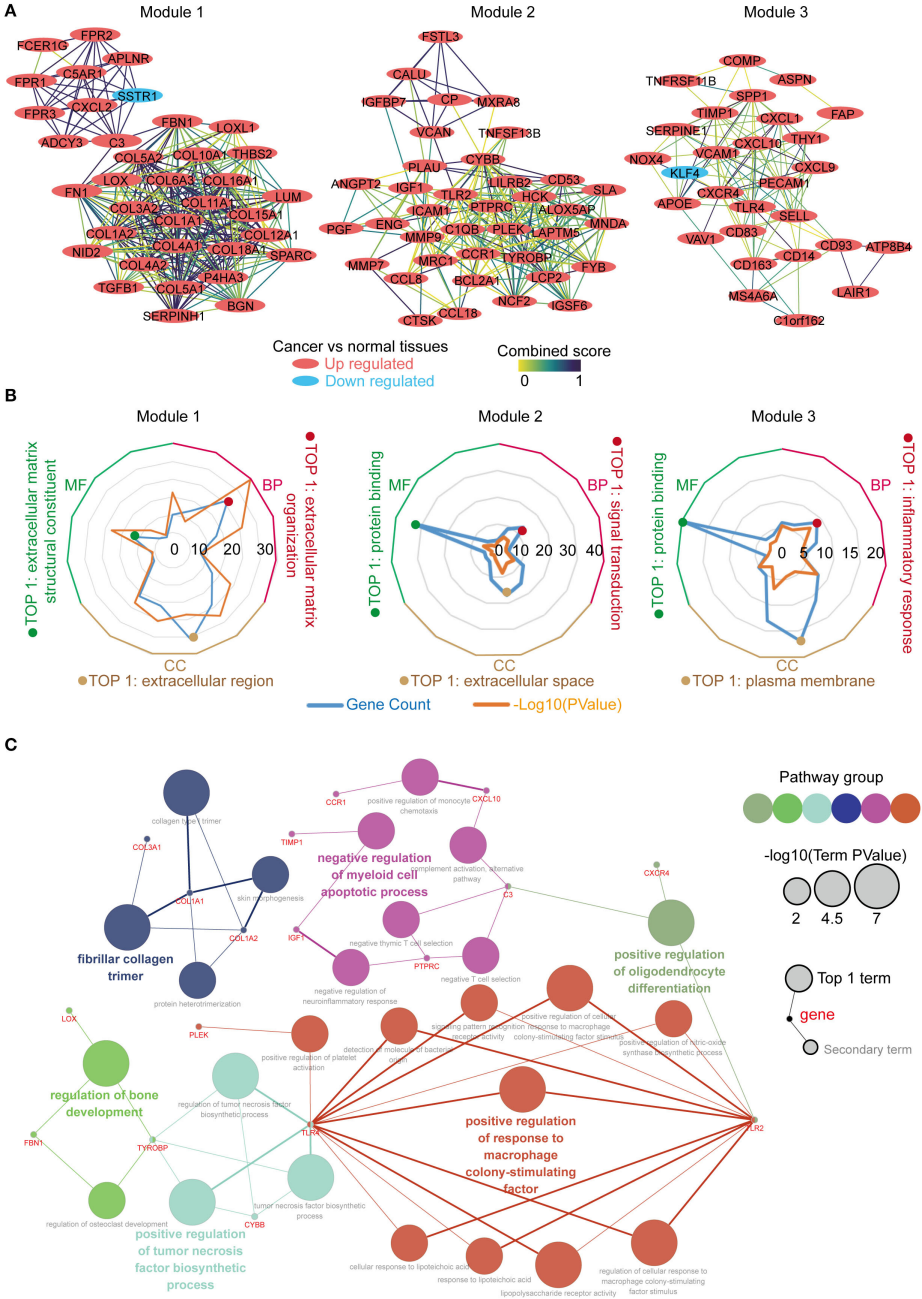
Identification, enrichment and interrelation analysis of the hub modules and the hub genes in PPI network. **(A)** Identification of the top 3 hub modules in the PPI network. **(B)** GO term enrichment analysis of the hub modules. **(C)** Interrelation analysis in the biological process pathways of the hub genes.

The PPI network was constructed using the STRING online database, and the top 3 significant modules were screened using the MCODE (Figure 3A). Module 1 contained 36 genes, with 35 upregulated and 1 down regulated in the tumor tissues vs. the normal tissues. Module 2 consisted of 38 genes, which were upregulated in the tumor tissues. Module 3 constituted 28 genes, with 27 upregulated and 1 downregulated in the tumor tissues. We performed function annotation of the modules using DAVID, as described previously. The GO analysis results (Figure 3B; Table S2) disclosed that module 1 was primarily enriched in the extracellular matrix organization, extracellular matrix structural constituent, and the extracellular region. Module 2, on the other hand, was primarily enriched in signal transduction, protein binding, and extracellular space, whereas module 3 was mainly involved in the inflammatory response, protein binding, and the plasma membrane. In addition, the top 30 hub genes with a high degree of connectivity in the PPI network were identified using cytoHubba (Supplementary Figure 1; Tables S3, S4). We performed an interrelation analysis between the pathways in the BPs of the hub genes using ClueGo to evaluate the pathway enrichment of the hub genes and the crosstalk between pathways. Consequently, the hub genes were primarily enriched in positive regulation of the response to macrophage colony-stimulating factor, positive regulation of the tumor necrosis factor biosynthetic process, negative regulation of the myeloid cell apoptotic process, and fibrillar collagen trimer (Figure 3C; Supplementary Figure 2). Based on the results above, we observed an enrichment of the hub modules and hub genes in the inflammatory response and extracellular matrix organization.

### Identification of the Hub Module and Hub Genes in WGCNA Network

**Figure 4:**
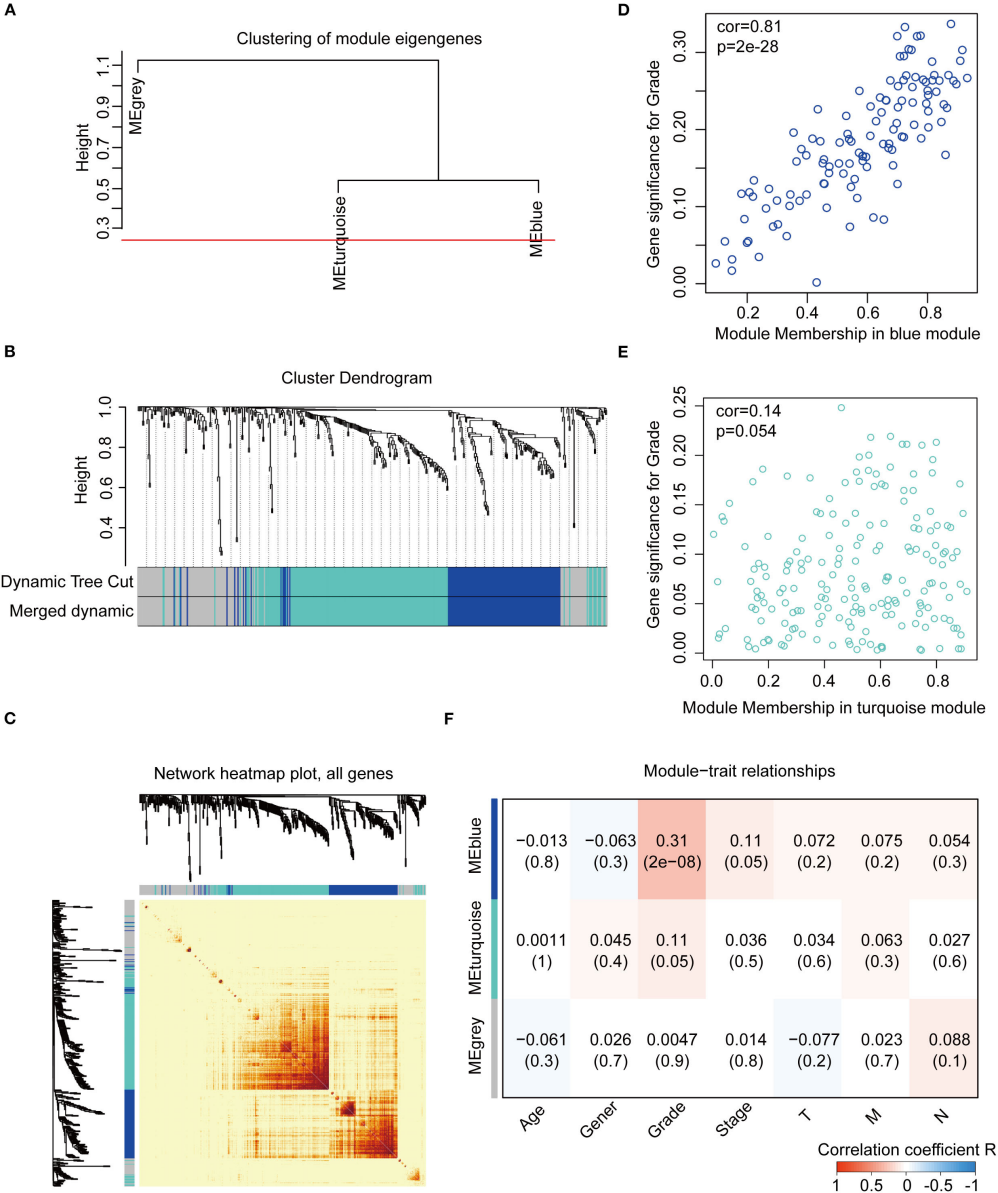
Construction and identification of the WGCNA co-expression modules associated with the clinical traits, based on the common DEGs expression data of TCGA-STAD. **(A)** Cluster dendrogram of the module eigengenes. The dissimilarity of module eigengenes is calculated to merge some similar modules with a height cut-off value of 0.25. **(B)** Cluster dendrogram of the DEGs. Highly similar modules are identified by clustering and then merged dynamically. **(C)** Network heatmap plot of the common DEGs. The branch in the hierarchical clustering dendrograms corresponds to each module. Color bars beneath and toward the right of the dendrograms show the color-coded module membership. The more saturated yellow and red indicate the higher co-expression interconnectedness in the heatmap. **(D)** Scatter plot of the GS for the grade vs. the MM in the blue module. **(E)** Scatter plot of the GS for the grade vs. the MM in the turquoise module. **(F)** Heatmap of the correlation between the module eigengenes and the clinical traits of gastric cancer. The blue module is the most positively correlated with the grade and identified as the hub module. GS, gene significance; MM, module membership.

We constructed the WGCNA network using the “WGCNA” R package. The expression patterns of the genes in the same module were similar and relevant by the average linkage clustering. We included 315 samples with clinical traits to filter the outlier samples via sample clustering of the common DEGs, and 17 samples were excluded with the height of 20,000 (Supplementary Figure 3A). A soft threshold (β) = 4 was set to ensure a scale-free network (*R*^2^ = 0.94; Supplementary Figures 3B–E). Similar modules with a height cut-off value of 0.25 were merged (Figure 4A), and 3 modules marked in blue, turquoise, and gray were identified (Figure 4B). The blue module contained 112 genes and the turquoise module 188 genes. Besides, 126 genes not included in any module were put into the gray module. The gray module was identified as not co-expressed and would be excluded in subsequent analyses. The interaction of the modules was visualized as the network heatmap (Figure 4C), which indicated that genes in the same module had a highly co-expressed relationship with each other. Then, the correlation between the GS and MM in the turquoise and blue modules was calculated, respectively. The correlation was significant in the blue module (*R* = 0.81, *P* < 0.001; Figure 4D) and not in the turquoise module (*R* = 0.14, *P* = 0.054; Figure 4E). Furthermore, the relationship between the modules and the clinical traits was evaluated to identify the hub module. The result showed that the blue module was significantly associated with the GC grade (*R* = 0.31, *P* < 0.001; Figure 4F). The top 30 hub genes in the blue module were screened using cytoHubba via the Degree method (Supplementary Figure 4; Table S5, Table S6). Consequently, the blue module was identified as the hub module in the WGCNA network and the top 30 hub genes in the blue module.

### Function Annotation, Pathway Enrichment, and Interrelation Analysis of the Blue Module and the Hub Genes in the WGCNA Network

**Figure 5:**
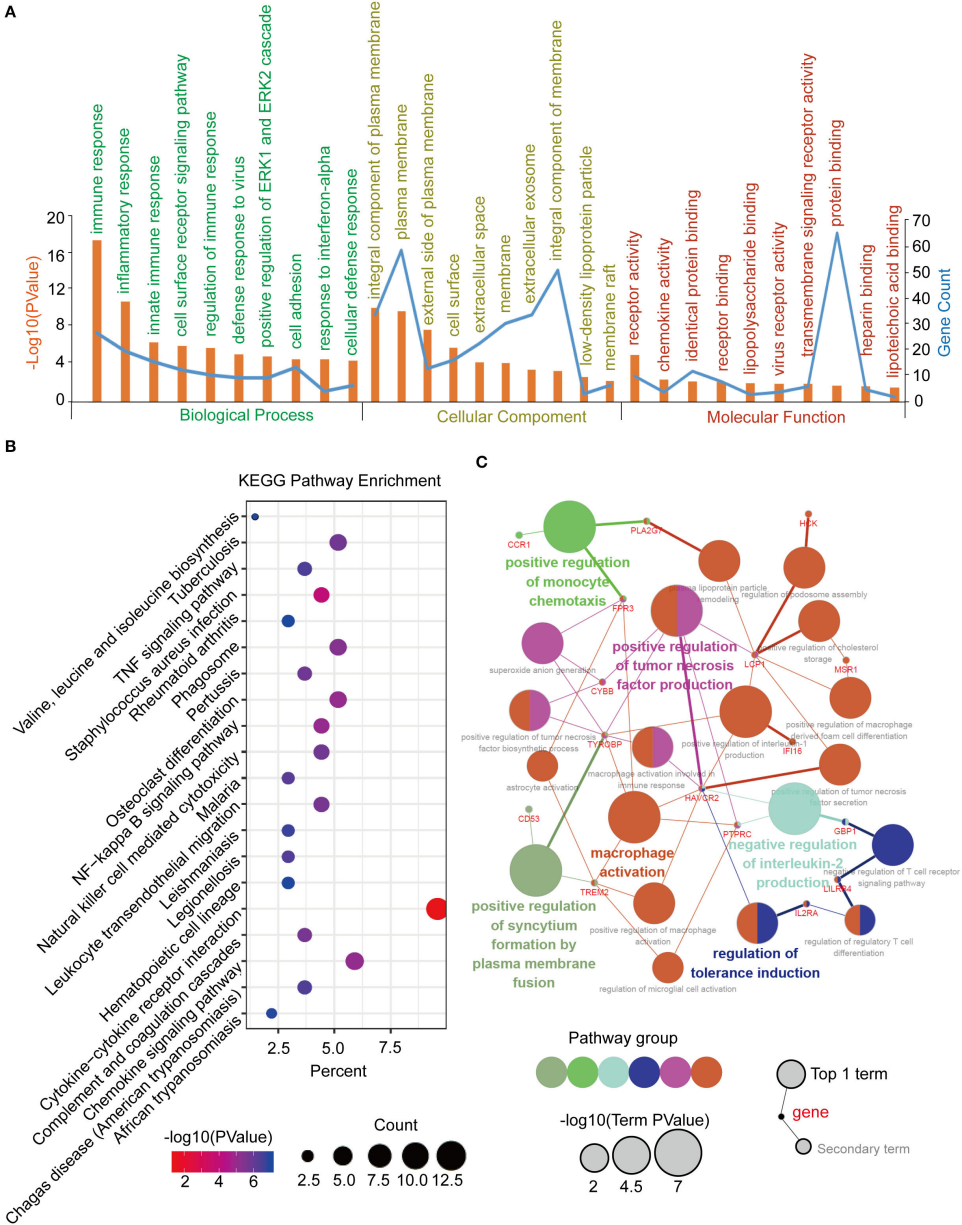
Function annotation and pathway enrichment of the hub module and hub genes in the WGCNA network. **(A)** GO analysis of the hub module in the WGCNA network. **(B)** KEGG pathway analysis of the hub module in the WGCNA network. **(C)** Interrelation analysis in the biological process pathways of the hub genes.

Function annotation and pathway enrichment of the blue module in the WGCNA network were performed in DAVID, as previously described. It was mainly enriched in the immune response and cell adhesion for BP, plasma membrane for CC, and protein binding for MF (Figure 5A). The KEGG pathway enrichment analysis identified the cytokine-cytokine receptor interaction as the most significantly enriched pathway, and the other pathways included the chemokine signaling pathway, tuberculosis, phagosome, and osteoclast differentiation (Figure 5B). Furthermore, an interrelation analysis between the pathways in the BPs of the hub genes was performed, as previously described. The hub genes were primarily involved in positive macrophage activation, regulation of tumor necrosis factor production, and regulation of tolerance induction (Figure 5C; Supplementary Figures 5A,B).

### Identification, Validation, and Survival Analysis of the Key Genes

**Figure 6:**
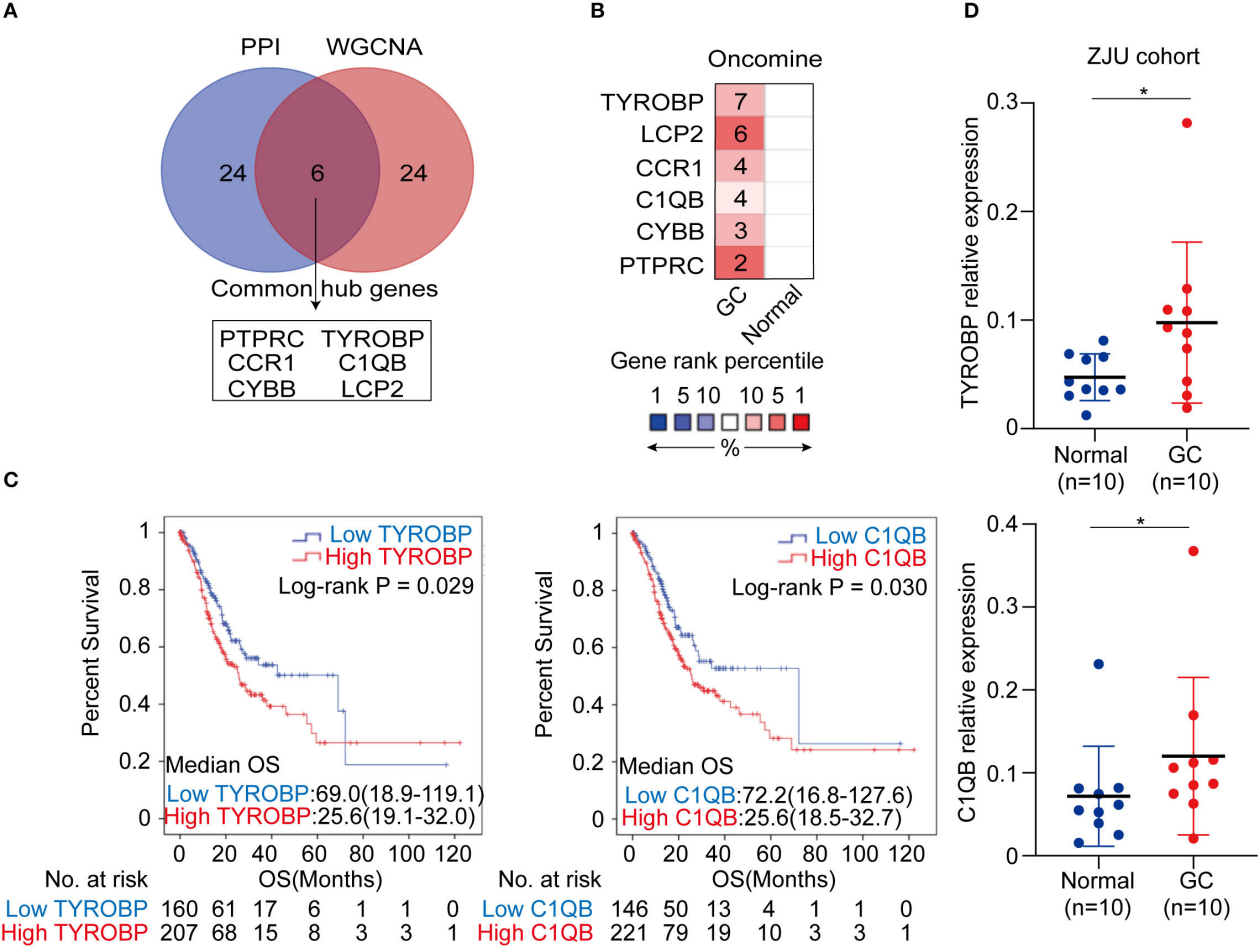
Identification, validation and survival analysis of key genes. **(A)** Identification of the common hub genes between the PPI network and the WGCNA network. **(B)** Validation of the common hub genes in ONCOMINE database. Red color represents relatively higher expression of the common hub genes in tumors than normal tissues. Numbers represented the number of studies. **(C)** Survival analysis of the key genes in TCGA-STAD dataset. **(D)** Validation of the key genes at the transcriptional level in ZJU cohort. *P < 0.05.

The top 30 hub genes in the PPI and WGCNA networks were overlapped to identify the common hub genes, including *PTPRC, TYROBP, CCR1, C1QB, CYBB*, and *LCP2* (Figure 6A). We then conducted a literature review to investigate the association between the hub genes in the networks and the tumors. Consequently, among the common hub genes, 50.0% (3/6) genes have been shown to promote tumor progression in gastric cancer. Among the other hub genes in the PPI and WGCNA networks, 70.8% (17/24) and 29.2% (7/24) have been reported in gastric cancer-associated studies, respectively (Table S7). We next focused on the common hub genes that might play a vital role in gastric cancer, considering the strong connection between the common hub genes and the other hub genes in the PPI and WGCNA networks. Based on the Oncomine database, using Data type = mRNA, *P*-value < 0.05, |FC| > 1.5 and gene rank = “all” as the threshold, the expression levels of the common hub genes were significantly higher in the GC tumor tissues compared with the normal or adjacent mucosa tissues (Figure 6B). Then, survival analyses of the common hub genes were performed using the TCGA-STAD dataset. The results showed that *TRYOBP* and *C1QB* were negatively associated with the overall survival of the GC patients (*P*_*TYROBP*_ = 0.029, *P*_*C*1*QB*_ = 0.030; Figure 6C), which was validated using the GSE15459 dataset (*P*_*TYROBP*_ = 0.001, *P*_*C*1*QB*_ = 0.001; Supplementary Figures 6A,B). However, *CCR1, CYBB, LCP2*, and *PTPRC* were not associated with the overall survival (*P*_*CCR*1_ = 0.412, *P*_*LCP*2_ = 0.148, *P*_*PTPRC*_ = 0.132, *P*_*CYBB*_ = 0.189; Supplementary Figures 7A–D). Based on these results, we further identified *TYROBP* and *C1QB* as the two key genes with prognostic value in gastric cancer. In addition, the high expression of *TYROBP* and *C1QB* were also validated in the GC tumor tissues compared with the adjacent mucosa tissues using the ZJU cohort (*P*_*TYROBP*_ = 0.045, *P*_*C*1*QB*_ = 0.031; Figure 6D). Besides, we explored the expression location of *TYROBP* and *C1QB* using the Human Protein Atlas database. As shown in the Supplementary Figure 8, *TYROBP* and *C1QB* were mainly located in tumor cells, which needed further validation in the experiments. Furthermore, we investigated the prognostic value of the other hub genes (the PPI or WGCNA network contained 24 genes) via univariate Cox analysis. We identified 50% (12/24) of the hub genes in the PPI network and 16.7% (4/24) of the hub genes in the WGCNA network that were negatively associated with the overall survival in GC patients (Table S8).

### The Correlation Between *TYROBP, C1QB*, and Immune Factors, Including Immune Cells and Biomarkers for Immunotherapy

**Figure 7:**
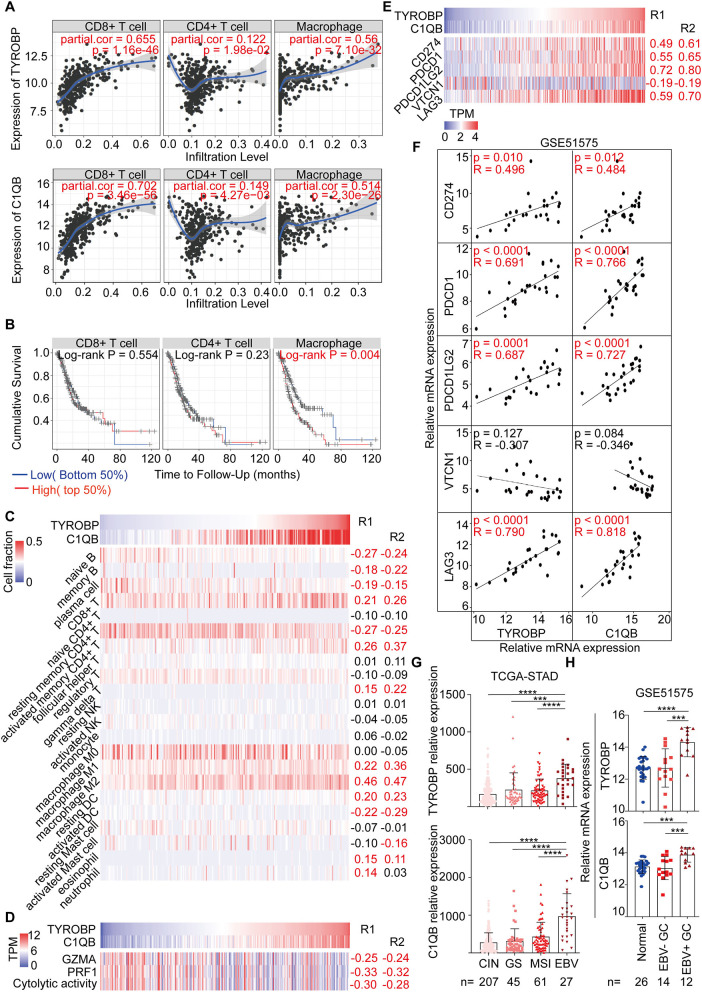
Correlation analysis between *TYROBP, C1QB*, and immune factors, including immune cells and biomarkers for immunotherapy. **(A)** Correlation between *TYROBP, C1QB*, and the immune cells in the TIMER database. **(B)** Survival analysis of the immune cells in the TIMER database. **(C)** Correlation between *TYROBP, C1QB*, and the immune cells in the TCGA-STAD dataset. **(D)** Correlation between *TYROBP, C1QB*, and the immune cytolytic activity in the TCGA-STAD dataset. **(E)** Correlation between *TYROBP, C1QB*, and the immune checkpoints in the TCGA-STAD dataset. **(F)** Correlation between *TYROBP, C1QB* and the immune checkpoints in the GSE51575 dataset. **(G)** Relative expression of *TYROBP* and *C1QB* in the four molecular subtypes in the TCGA-STAD dataset. **(H)** Relative expression of *TYROBP* and *C1QB* in normal tissues, EBV negative (–) and positive (+) GC tissues in the GSE51575 dataset. R1, the correlation coefficient between *TYROBP* and the immune factors; R2, the correlation coefficient between *C1QB* and the immune factors; GC, gastric cancer. The data marked red color is statistically significant. ***P < 0.001, ****P < 0.0001.

As mentioned, we found that *TYROBP* and *C1QB* could be involved in the immune response via the function annotation, pathway enrichment, and interrelation analysis. Therefore, we evaluated the correlation between *TYROBP, C1QB*, and immune factors, respectively. The TIMER database analysis results revealed a significantly positive correlation of both genes with the CD8^+^ T cells, CD4^+^ T cells, and macrophages (Figure 7A). Survival analyses of the immune cells were also performed using the TIMER database, and the results indicated that macrophages were negatively associated with the survival time of GC patients (*P* = 0.004; Figure 7B). Furthermore, using the CIBERSORT algorithm, we estimated the proportion of the infiltrating immune cells in the tumor microenvironment. Consequently, we found that the infiltrating immune cells mainly consisted of the CD8^+^ T cells, CD4^+^ T cells, and macrophages. *TYROBP* and *C1QB* were negatively correlated with resting memory CD4^+^ T cells (R1 = −0.27, R2 = −0.25; Figure 7C; Table S9) and positively correlated with the CD8^+^ T cells (R1 = 0.21, R2 = 0.26; Figure 7C; Table S9), activated memory CD4^+^ T cells (R1 = 0.26, R2 = 0.37; Figure 7C; Table S9), and macrophage M2 (R1 = 0.46, R2 = 0.47; Figure 7C; Table S9). We performed correlation analyses for *TYROBP* and *C1QB* with differential markers of macrophages to further investigate the association of *TYROBP* and *C1QB* with the macrophages. As shown in the Supplementary Figure 9, the correlation of CD11b and CD206 (M2) was stronger compared with the CD68 (M0) and CCR7 (M1). Furthermore, we performed univariate and multivariate Cox regression for *TYROBP, C1QB*, and macrophages, respectively. As shown in Table S10, *TYROBP* (HR = 1.455, *P* = 0.029), *C1QB* (HR = 1.474, *P* = 0.030), and macrophage M2 (HR = 1.494, *P* = 0.024) were identified as the significant risk factors, while macrophage M1 (HR = 1.182, *P* = 0.374) was not associated with the overall survival via the univariate Cox regression analysis. Notably, in the multivariate Cox regression model, both *TYROBP* (HR = 1.399, *P* = 0.073) and *C1QB* (HR = 1.428, *P* = 0.067) were not significantly correlated with the overall survival when adjusted by macrophage M2. However, *TYROBP* (HR = 1.518, *P* = 0.023) and *C1QB* (HR = 1.550, *P* = 0.024) were still significantly associated with poor outcomes when adjusted by macrophage M1. These findings suggested that macrophage M2 is involved in *TYROBP/C1QB*-mediated progression and poor survival outcomes in GC. Then, the cytolytic activity of the immune cells was estimated using the average expression of *GZMA* and *PRF1* (27). The results showed that *TYROBP* and *C1QB* were negatively correlated with the cytolytic activity (R1 = −0.30, R2 = −0.28; Figure 7D; Table S9), which revealed the immunosuppressive microenvironment in tumors. Furthermore, the correlation between *TYROBP, C1QB*, and immune checkpoints was assessed, respectively. Consequently, *TYROBP* and *C1QB* were positively correlated with *CD274, PDCD1, PDCD1LG2*, and *LAG3* but negatively correlated with *VTCN1* in the TCGA-STAD dataset (Figure 7E; Table S9) and GSE51575 dataset (Figure 7F). In addition, we evaluated the expression of *TYROBP* and *C1QB* in the TCGA subtypes. Compared with the other subtypes, the expression of *TYROBP* and *C1QB* in the EBV positive subtype was significantly higher (*P*_*TYROBP*_ < 0.0001, *P*_*C*1*QB*_ < 0.0001; Figure 7G). These results were validated using the GSE51575 dataset (*P*_*TYROBP*_ < 0.0001, *P*_*C*1*QB*_ = 0.0002; Figure 7H). In summary, we established that the expression of *TYROBP* and *C1QB* was positively correlated with the PD-L1 expression, CD8^+^ T cell infiltration, and the EBV status; three predictive biomarkers for immunotherapy.

## Discussion

GC is the third leading cause of global cancer-related deaths. However, to date, effective treatments have not yet been developed, owing to a limited understanding of the molecular mechanisms underlying GC development. Over the past years, the applications of PD-1/PD-L1 checkpoint blockades in cancer have revolutionized oncology (28, 29). Particularly, these approaches have guided immunotherapy strategies against multiple cancers, such as melanoma (30), lung cancer (31), glioblastoma (32) and liver cancer (33). However, the efficacy and responsiveness of immunotherapeutic agents significantly varies across GC patients, largely due to high tumor heterogeneity and molecular complexity (34). Thus, it is crucial to unravel the underlying molecular mechanisms of GC tumorigenesis and progression and identify potential prognostic and therapeutic targets. In the present study, analysis of four gene expression profiles revealed common DEGs, mainly enriched in signal transduction, cell adhesion and immune response. Previous studies have shown that extracellular matrix remodeling and abnormal immune microenvironment play important roles in tumorigenesis and tumor progression. In the past decade, studies have demonstrated that the interaction between tumor microenvironment and tumor cells is essential for tumor biological behavior (35–38). In the present study, PPI and WGCNA networks revealed 6 common hub genes, including *PTPRC, TYROBP, CCR1, C1QB, CYBB*, and *LCP2*. According to Wang et al. (39), *CYBB* is associated with invasion and prognosis of human gastric cancer, whereas *PTPRC*, also known as CD45, has been previously used to assess the extent of immune cell infiltration in intestinal-type Japanese gastric cancer (40). On the other hand, Chen et al. (41) previously reported that *CCR1* was associated with CD4^+^CD25^+^ Tregs of regional lymph nodes in forestomach carcinoma. Interestingly, *TYROBP* and *C1QB* were both correlated with immune infiltration levels, suggesting a potential key role in prognosis of GC patients. These factors have previously been positively associated with three predictive biomarkers for immunotherapy in GC, including PD-L1 expression (42, 43), CD8^+^ T cells infiltration (44) and EBV status (45, 46).

Previous studies have shown that *TYROBP*, also known as *DAP12*, is overexpressed and related to tumor progression in multiple cancers. Functionally, its encoded protein, a transmembrane signaling polypeptide on the surface of a variety of immune cells, mediates signaling transductions (47, 48). For example, Shabo et al. (49) reported an association between high *TYROBP* expression with skeletal and liver metastases as well as poor survival of breast cancer patients. Similarly, Cheray et al. (50) implicated *TYROBP* in glioblastoma tumorigenesis and aggressiveness. In the present study, *TYROBP* overexpression was associated with poor survival of GC patients. In addition, results from interrelation analysis showed that *TYROBP* was associated with positive macrophage activation, regulation of tumor necrosis factor production and regulation of tolerance induction. This is consistent with a previous study that found a positive association between *TYROBP* with macrophage M2, as well as the immunosuppressive and pro-tumorigenic subtype of macrophage in the tumor microenvironment (51). Similarly, Takamiya et al. (52) found that *TYROBP* was involved in the interaction between lung cancer cell and macrophage M2 to enhance TGF-β secretion *in vitro*. Our results further revealed a positive correlation between *TYROBP* and CD8^+^ T cells, but a negative association with cytolytic activity. In addition, we found a positive association between *TYROBP* with most checkpoints, including *CD274, PDCD1, PDCD1LG2*, and *LAG3*. Taken together, these results indicated that *TYROBP* might be playing an immunosuppressive role on CD8^+^ T cells and macrophages to promote tumor immune escape in gastric cancer. Coincidentally, Yoshida et al. (53) reported that *TYROBP* deficiency in liver allografts resulted in activation of graft-infiltrating CD8^+^ T cells and production of pro-inflammatory cytokine, whereas Kovats et al. (54) found that loss of *TYROBP* and FcRγ promoted IL-12 production and CD8^+^ T cell response by CCR2^+^ Mo-DCs. Thus, *TYROBP* might be a negative factor in anti-tumor immune response. Furthermore, we found a significantly higher *TYROBP* expression in EBV positive patients relative to those with other subtypes. To date, EBV status is one of the validated predictive biomarkers for immunotherapy (45, 46). These results suggest that *TYROBP* might be associated with the multiple biomarkers for immunotherapy in gastric cancer, although further validation using large clinical cohorts is required.

In the present study, we found significantly higher expression of *C1QB*, that encodes the C1qB chain, in tumor than adjacent normal GC tissues. In addition, *C1QB* was negatively associated with prognosis of GC patients. Previous studies have shown that C1q, the first recognition subcomponent of the complement classical pathway, comprises three chains (C1qA, C1qB, and C1qC) and exerts complex effects on tumorigenesis of multiple tumors, including prostate (55), and ovarian cancer (56) as well as gliomas (57). Yamada et al. (58) reported that high *C1QB* expression was significantly related to poor prognosis in renal cell carcinoma. On the other hand, Linnartz-Gerlach et al. (59) found that C1qB was downregulated in the brain of triggering receptor expressed on myeloid cells-2 (*TREM2*) knock-out mice. Interestingly, *TREM2* has been reported to transmit intracellular signals through the associated transmembrane adapter *TYROBP* (60). In the present study, we found a strong correlation between *TYROBP* and *C1QB* expression in GC patients (*R* = 0.92, *P* < 0.001; Supplementary Figure 10). Studies have shown that a dysregulation of this signaling pathway leads to a wide range of pathophysiological changes and diseases, such as aging (59), bone cysts (61) and Alzheimer's disease (62). In our study, we also found an association between *C1QB* with PD-L1 expression, CD8^+^ T cells infiltration and EBV status, which was very similar to the *TYROBP* pattern. However, *in vitro* and *in vivo* studies are needed to validate the observed relationship between *TYROBP* and *C1QB* in GC patients.

This study had some limitations. Firstly, our results will be more convincing and interesting through additional validation of *TYROBP* and *C1QB in vivo in vitro* experiment. For example, immunofluorescent detection is more precise than immunohistochemistry for analyzing co-localization of *TYROBP* and *C1QB*. Secondly, although our integrated network analysis indicated the prognosis value of *TYROBP* and *C1QB* in gastric cancer, further validation is needed using more clinical cohorts. Importantly, our bioinformatics findings indicated that macrophage M2 might be involved in *TYROBP*/*C1QB*-mediated progression and poor survival outcomes in GC. Further experimental studies are needed to unravel the role of macrophage M2 in GC.

In conclusion, we used integrated network analysis, PPI and WGCNA, to reveal overexpression of *TYROBP* and *C1QB*, and affirm their prognostic value in GC patients. To our knowledge, this is the first report associating *TYROBP* and *C1QB* with GC progression and prognosis. Our findings lay a foundation for future research aiming to elucidate the role of these genes in GC tumorigenesis and progression.

## Data Availability Statement

Publicly available datasets were analyzed in this study, these can be found in The Cancer Genome Atlas (https://portal.gdc.cancer.gov/); the NCBI Gene Expression Omnibus (GSE65801, GSE54129, GSE15459, GSE51575, and GSE118916).

## Ethics Statement

The studies involving human participants were reviewed and approved by Research Ethics Committee of the First Affiliated Hospital, College of Medicine, Zhejiang University. The patients/participants provided their written informed consent to participate in this study.

## Author Contributions

LT conceived and designed the study. JJ and YD collected data, performed data analysis, and wrote manuscript. MW, YC, HaifW, and HaiyW were involved in data interpretation and critically reviewed the manuscript. XL performed qRT-PCR. All authors read and approved the final manuscript.

## Conflict of Interest

The authors declare that the research was conducted in the absence of any commercial or financial relationships that could be construed as a potential conflict of interest.
