# Supplementary material for: Identification of TYROBP and C1QB as Two Novel Key Genes With Prognostic Value in Gastric Cancer by Network Analysis
Source: Front Oncol. 2020 Sep 11;10:1765. doi: 10.3389/fonc.2020.01765 (PMC7516284; doi:10.3389/fonc.2020.01765)
Supplement: Supplementary file 1 [file Data_Sheet_1.pdf]

Supplementary Table

Table S1 333 up-regulated and 93 down-regulated genes were identified from TCGA-STAD, GSE65801, GSE54129 and GSE118916

DEGs	Gene Name
Up - regulated	*CXCR4, MMP7, CDC25B, SLCO2B1, COL1A1, CST7, SELL, PLEKHG4, FNDC1, SAMSN1COL18A1, FUS, ASS1, CD53, TFEC, BICD1, DUSP10, CR1, FPR1, ARHGDIB, F2RL2, LRRC32, ALOX5AP, SH3PXD2B, ITGBL1, CPXM1, LRP8, COMP, WISP1, SLC15A3, ADORA2B, SLFN11, ADAP2, SULF1, SLC39A10, RARRES1, IGSF6, COL11A1, TREM1, GBP5, SERPINA3, C1QC, SH3RF3, GPNMB, CD55, FZD2, TNFRSF11B, IGFBP7, LTBP2, MAP3K8, HLA_DMB, EGFLAM, IER5L, TNFRSF12A, DFNA5, PDPN, COL3A1, PECAM1, IFITM3, CCR1, SERPINE2, NOD1, CCL8, GPR34, SLC20A1, AEBP1, PCDHB16, CXCL1, FAM43A, HSPH1, SIGLEC7, FJX1, PHTF2, SLC7A6, IGF1, PRR16, SERPINB9, LAIR1, C3, PIK3AP1, DLGAP4, FN1, IFI16, RASSF4, CTSB, ATP8B4, SPP1, CTSK, LST1, CDK4, MXRA5, RASGEF1A, GXYLT2, RGS1, PLXND1, PTPRC, SDS, CNN2, COL4A2, LUM, HHEX, THBS4, FSCN1, SRPX2, CLEC7A, CYBB, NID2, PDE4B, LCP2, GLS, EMILIN2, LOX, IL32, LCP1, CLDN1, FCER1, ELK3, IFITM1, HAVCR2, TMEM176B, MCTP1, DPYSL2, FPR3, NRP1, PLEK, TMEM45A, PLA2G2A, PLXNC1, MNDA, CPNE2, SLC19A3, MYOF, PCDHB2, NOX4, SFRP4, GRB14, CCDC8, ENG, TYROBP, TMEM158, DAB2, SERPINH1, IL2RA, FAP, TNFSF4, INHBA, CALU, TNFSF13B, TMEM173, MDK, CALCRL, ICAM1, THBS2, ETS1, SERPINE1, LY6E, CSF2RA, COL10A1, BCAT1, TGFBI, C5AR1, APOE, TUBB, TM6SF1, CMTM3, OSR2, SFRP2, TNFAIP6, KIAA0226L, ADCY3, SLA, CPVL, PI15, CD83, SULF2, CD93, PODXL, CDH11, BGN, COCH, TNFAIP3, ASPN, CCL18, TREM2, THSD7A, CXCL2, CHRDL2, HOXC10, MCM2, CETP, CXCL10, VCAN, AJUBA, FPR2, COL1A2, C1orf162, COL12A1, ESM1, RCN3, TRIO, CHSY1, PTPN12, MXRA8, FAS, OSMR, CCT2, SLCO3A1, NCF2, CST1, SNX10, CLDN7, MS4A6A, IFITM2, GJA4, FKBP10, DDAH2, C10orf10, GREM1, BUB1, CD163, GPR65, BST2, FAM198B, HOXC9, FADS3, TNFRSF10B, HIF1A, C1QB, S100A10, TLR4, GLIPR1, COL6A3, PLEKHA4, CLEC4A, TDO2, SPARC, COL5A1, CTHRC1, PLXDC1, E2F3, FGD6, TPX2, LILRB2, IGF2BP3, ANP32E, LAMP3, P4HA3, FYB, MAPRE1, KDELC1, CHI3L1, LOXL1, PRRX1, HCK, ASAP1, COL5A2, THY1, TMEM176A, CD200, MSR1, TIMP1, MRC1, NNMT, BCL2A1, ECT2, CD14, TRIP6, CDH3, PGF, WNT2, CSGALNACT1, GBP1, C2, ARPC1B, PLA2G7, MFAP2, COL16A1, GPX8, PCDH17, COL4A1, ALDH1A3, ENTPD1, CPNE1, HAPLN3, CTSA, TLR2, AMIGO2, MYO1B, PLAU, CDH13, RNASE6, TGM2, RFTN1, COL15A1, LILRB4, FOXC1, ABCA1, ADAMTS9, RAI14, HOXB3, APLNR, IL13RA2, HLA_DPA1, LTB, FMO1, FBN1, INHBB, S100A11, ANGPT2, CPNE8, ST3GAL2, SACS, S100A4, EPB41L2, PLXDC2, HOMER1, CXCL9, MARCH1, OLFML2B, VCAM1, PPFIA1, LAPTM5, FSTL3, PMEPA1, HOXC6, HES4, NPL, CP, VAV1, MMP9, SLC39A6, CTSC*
Down - regulated	*MAOA, PMM1, GKN1, ACADL, ENHO, NRG4, KLF4, MICALL1, TNFRSF17, CYP4F12, SCNN1B, ZBTB7C, GGT6, UBE2QL1, MT1E, ANXA10, GIF, NME5, GDPD2, SOSTDC1, FAM46C, SH3GL2, CKMT2, KCNJ16, GKN2, AKR1B10,*

	PTPRZ1, ATP4A, UGT2B15, MAL, ESRRG, AMPD1, TFF2, MT1X, SPINK2, LIPF, CAPN13, CYP2C18, FUT9, C16orf89, PGC, AKR1C3, SELENBP1, CBR1, NTPD3, AKR7A3, FAM3B, ADH1C, PLCXD3, CWH43, GPT, CXCL17, NR3C2, KRT20, RNASE1, MYZAP, AKR1C1, VSIG2, SCNN1G, PSAPL1, PSCA, SPTSSB, TFF1, DERL3, LDHD, FBP2, AADAC, FAM150B, SCIN, CAPN9, VILL, ADH7, CCKBR, ADRB2, HPGD, SCGB2A1, GUCA2B, ETFDH, LIFR, ALDH3A1, RDH12, CA2, LTF, GSTA3, MFSD4, SSTR1, PAIP2B, KCNE2, SIGLEC11, ALDOB, TM6SF2, KCNJ15, SULT1B1

Table S2 GO term enrichment analysis of hub modules from PPI network in gastric cancer

	Category	Term	Count	-log10(PValue)
Module 1	BP	GO:0030198~extracellular matrix organization	21	29.64
	BP	GO:0030574~collagen catabolic process	13	20.61
	BP	GO:0030199~collagen fibril organization	10	16.31
	BP	GO:0007155~cell adhesion	12	8.88
	BP	GO:0050900~leukocyte migration	8	8.29
	CC	GO:0031012~extracellular matrix	19	22.89
	CC	GO:0005788~endoplasmic reticulum lumen	16	20.47
	CC	GO:0005581~collagen trimer	13	19.01
	CC	GO:0005576~extracellular region	26	18.55
	CC	GO:0005578~proteinaceous extracellular matrix	16	18.27
	MF	GO:0005201~extracellular matrix structural constituent	12	18.20
	MF	GO:0048407~platelet-derived growth factor binding	5	8.30
	MF	GO:0005518~collagen binding	6	6.84
	MF	GO:0005178~integrin binding	6	5.63
	MF	GO:0050840~extracellular matrix binding	4	4.69
Module 2	BP	GO:0006955~immune response	9	5.62
	BP	GO:0007165~signal transduction	13	5.53
	BP	GO:0006968~cellular defense response	4	3.50
	BP	GO:0001666~response to hypoxia	5	3.31
	BP	GO:0022617~extracellular matrix disassembly	4	3.24
	CC	GO:0005615~extracellular space	17	8.68
	CC	GO:0009986~cell surface	11	7.07
	CC	GO:0005576~extracellular region	15	5.84
	CC	GO:0005887~integral component of plasma membrane	11	3.41
	CC	GO:0005925~focal adhesion	5	2.10
	MF	GO:0005515~protein binding	33	5.53
	MF	GO:0004888~transmembrane signaling receptor activity	5	2.97
	MF	GO:0004252~serine-type endopeptidase activity	5	2.69
	MF	GO:0005102~receptor binding	5	2.18
	MF	GO:0004872~receptor activity	4	1.96
Module 3	BP	GO:0006954~inflammatory response	9	7.32

BP	GO:0032496~response to lipopolysaccharide	6	5.42
BP	GO:0030198~extracellular matrix organization	6	5.04
BP	GO:0007155~cell adhesion	7	4.34
BP	GO:0060326~cell chemotaxis	4	3.92
CC	GO:0009897~external side of plasma membrane	8	7.71
CC	GO:0005615~extracellular space	13	6.81
CC	GO:0005886~plasma membrane	17	4.36
CC	GO:0005576~extracellular region	11	4.23
CC	GO:0031012~extracellular matrix	5	3.05
MF	GO:0002020~protease binding	5	4.93
MF	GO:0008201~heparin binding	4	2.84
MF	GO:0005125~cytokine activity	4	2.72
MF	GO:0008009~chemokine activity	3	2.66
MF	GO:0005515~protein binding	20	2.23

Table S3 Top 30 nodes in PPI network by Degree method

Gene Rank	Node	Rank Score	Fold Change	Gene Rank	Node	Rank Score	Fold Change
1	*FN1*	25	1.91	14	*CXCL1*	16	1.91
2	*MMP9*	24	5.01	17	*CYBB*	15	5.01
2	*TLR4*	24	1.97	17	*LOX*	15	1.97
4	*PTPRC*	23	1.97	19	*CD163*	13	1.97
4	*VCAM1*	23	2.35	19	*C1QB*	13	2.35
6	*TLR2*	22	3.51	19	*LCP2*	13	3.51
7	*ICAM1*	21	3.07	19	*PLEK*	13	3.07
7	*SPP1*	21	23.95	19	*C3*	13	23.95
7	*CCR1*	21	2.74	19	*BGN*	13	2.74
10	*PECAM1*	20	1.50	25	*COL1A1*	12	1.50
11	*TIMP1*	19	4.23	25	*TYROBP*	12	4.23
11	*CXCR4*	19	1.82	27	*LILRB2*	11	1.82
13	*SELL*	17	2.03	28	*COL1A2*	10	2.03
14	*IGF1*	16	1.83	28	*COL3A1*	10	1.83
14	*CXCL10*	16	13.14	28	*FBN1*	10	13.14

Table S4 Edges in PPI network by Degree method

Node1	Node2	Score	Node1	Node2	Score	Node1	Node2	Score
COL1A2	*COL1A1*	0.999	*VCAM1*	*SELL*	0.744	*CXCL10*	*TIMP1*	0.568
MMP9	*TIMP1*	0.997	*COL3A1*	*LOX*	0.743	*VCAM1*	*TIMP1*	0.568
COL3A1	*COL1A2*	0.995	*MMP9*	*CAM1*	0.737	*MMP9*	*CD163*	0.566
COL3A1	*COL1A1*	0.994	*CXCL10*	*TLR2*	0.734	*LILRB2*	*PLEK*	0.562
PTPRC	*CXCR4*	0.99	*MMP9*	*COL1A1*	0.733	*VCAM1*	*PLEK*	0.562
FN1	*FBN1*	0.989	*CXCL10*	*ICAM1*	0.732	*CXCR4*	*IGF1*	0.561
CXCL1	*CL10*	0.987	*SPP1*	*IGF1*	0.732	*PECAM1*	*SPP1*	0.56
CXCL1	*MMP9*	0.983	*CXCR4*	*TLR2*	0.723	*VCAM1*	*TLR2*	0.56
TLR4	*BGN*	0.981	*PTPRC*	*TLR4*	0.72	*MMP9*	*COL3A1*	0.558
SPP1	*TIMP1*	0.978	*SPP1*	*ICAM1*	0.719	*CXCR4*	*SPP1*	0.556
SPP1	*FN1*	0.977	*LILRB2*	*CD163*	0.717	*CXCL1*	*FN1*	0.555
TYROBP	*LCP2*	0.977	*CXCR4*	*SELL*	0.714	*SELL*	*LCP2*	0.554
CXCL10	*CCR1*	0.972	*COL1A1*	*TIMP1*	0.713	*CCR1*	*SELL*	0.553
FN1	*TIMP1*	0.972	*CCR1*	*LCP2*	0.707	*FN1*	*SELL*	0.553
PTPRC	*LCP2*	0.969	*PECAM1*	*SELL*	0.703	*LILRB2*	*CCR1*	0.553
FN1	*IGF1*	0.968	*PTPRC*	*ICAM1*	0.702	*PECAM1*	*CXCL1*	0.552
FN1	*C3*	0.966	*IGF1*	*COL1A2*	0.701	*CYBB*	*CCR1*	0.546
CXCR4	*CXCL10*	0.965	*PTPRC*	*CD163*	0.699	*CCR1*	*VCAM1*	0.545
CXCR4	*CXCL1*	0.964	*MMP9*	*VCAM1*	0.696	*VCAM1*	*LCP2*	0.544
CXCL1	*CCR1*	0.96	*PECAM1*	*MMP9*	0.696	*TLR2*	*C3*	0.541
BGN	*TLR2*	0.955	*SPP1*	*COL1A1*	0.693	*TLR4*	*C3*	0.541
CYBB	*VCAM1*	0.954	*CCR1*	*ICAM1*	0.692	*LILRB2*	*LCP2*	0.54
FBN1	*COL3A1*	0.953	*PECAM1*	*TLR4*	0.689	*SPP1*	*COL1A2*	0.539
IGF1	*TIMP1*	0.953	*CXCL10*	*VCAM1*	0.687	*BGN*	*ICAM1*	0.538
LILRB2	*CYBB*	0.944	*CXCR4*	*VCAM1*	0.687	*FN1*	*C1QB*	0.524
PECAM1	*PTPRC*	0.941	*TLR4*	*VCAM1*	0.687	*CYBB*	*C1QB*	0.514
VCAM1	*ICAM1*	0.94	*ICAM1*	*TLR2*	0.685	*CYBB*	*LCP2*	0.511
FBN1	*TIMP1*	0.939	*IGF1*	*ICAM1*	0.684	*IGF1*	*COL1A1*	0.509
CXCL1	*C3*	0.931	*CXCR4*	*FN1*	0.683	*PECAM1*	*TIMP1*	0.509
CXCL10	*C3*	0.931	*PTPRC*	*TLR2*	0.682	*CXCL1*	*SELL*	0.504
SPP1	*C3*	0.93	*MMP9*	*COL1A2*	0.68	*TYROBP*	*SELL*	0.503
LILRB2	*TYROBP*	0.926	*COL1A2*	*TIMP1*	0.677	*ICAM1*	*C3*	0.502
SPP1	*FBN1*	0.926	*PTPRC*	*CCR1*	0.676	*CXCL1*	*SPP1*	0.501
C3	*TIMP1*	0.921	*TLR4*	*IGF1*	0.674	*MMP9*	*SELL*	0.501
CXCR4	*C3*	0.921	*BGN*	*FBN1*	0.67	*IGF1*	*VCAM1*	0.494
CXCR4	*CCR1*	0.921	*CXCR4*	*ICAM1*	0.664	*C1QB*	*C3*	0.487
FBN1	*COL1A2*	0.921	*CYBB*	*TLR2*	0.661	*MMP9*	*BGN*	0.487
CCR1	*C3*	0.914	*COL3A1*	*TIMP1*	0.658	*IGF1*	*PLEK*	0.481
PTPRC	*SELL*	0.912	*CXCL1*	*VCAM1*	0.658	*PECAM1*	*LOX*	0.48
FBN1	*C3*	0.906	*TLR4*	*FN1*	0.648	*TLR4*	*TIMP1*	0.474
CYBB	*MMP9*	0.903	*TLR4*	*PLEK*	0.647	*MMP9*	*FBN1*	0.472
TLR4	*TLR2*	0.902	*PTPRC*	*TYROBP*	0.644	*SELL*	*PLEK*	0.472
FN1	*LOX*	0.898	*PTPRC*	*CYBB*	0.642	*CCR1*	*PLEK*	0.47
FN1	*COL1A2*	0.893	*SPP1*	*CCR1*	0.641	*PECAM1*	*CXCL10*	0.469
BGN	*COL1A2*	0.891	*TLR4*	*C1QB*	0.641	*CYBB*	*FN1*	0.468
C1QB	*TYROBP*	0.89	*PECAM1*	*CD163*	0.636	*C1QB*	*CCR1*	0.467

BGN	*COL1A1*	0.873	*TLR4*	*CD163*	0.636	*PECAM1*	*COL1A1*	0.466
PLEK	*LCP2*	0.871	*TLR4*	*TYROBP*	0.628	*CD163*	*VCAM1*	0.464
PTPRC	*VCAM1*	0.871	*TLR2*	*SELL*	0.627	*SPP1*	*TLR4*	0.459
FN1	*COL1A1*	0.869	*CYBB*	*PLEK*	0.625	*IGF1*	*LOX*	0.458
CD163	*C1QB*	0.85	*MMP9*	*IGF1*	0.621	*SPP1*	*LOX*	0.458
FN1	*COL3A1*	0.848	*CXCL1*	*TIMP1*	0.62	*VCAM1*	*LOX*	0.457
TLR4	*CXCL10*	0.84	*PECAM1*	*LCP2*	0.62	*CD163*	*CCR1*	0.456
TLR4	*ICAM1*	0.836	*ICAM1*	*TIMP1*	0.619	*CD163*	*ICAM1*	0.455
FBN1	*COL1A1*	0.835	*TLR4*	*LCP2*	0.619	*PECAM1*	*CYBB*	0.453
TYROBP	*TLR2*	0.835	*PTPRC*	*CXCL10*	0.617	*CXCR4*	*CD163*	0.451
TLR4	*MMP9*	0.834	*TLR4*	*SELL*	0.617	*SPP1*	*CXCL10*	0.451
CD163	*TYROBP*	0.827	*PTPRC*	*LILRB2*	0.61	*CYBB*	*SELL*	0.45
LILRB2	*TLR2*	0.822	*PTPRC*	*CXCL1*	0.609	*ICAM1*	*LCP2*	0.45
FBN1	*LOX*	0.82	*CCR1*	*TYROBP*	0.602	*FN1*	*CXCL10*	0.448
LOX	*COL1A1*	0.82	*CXCR4*	*TLR4*	0.602	*PECAM1*	*TLR2*	0.446
CYBB	*TLR4*	0.819	*SPP1*	*BGN*	0.602	*TLR2*	*PLEK*	0.446
FN1	*ICAM1*	0.819	*MMP9*	*FN1*	0.599	*PTPRC*	*COL1A1*	0.444
PECAM1	*VCAM1*	0.818	*PECAM1*	*IGF1*	0.599	*CXCR4*	*IMP1*	0.44
SPP1	*MMP9*	0.818	*CD163*	*CXCL10*	0.598	*SPP1*	*TLR2*	0.44
CXCL1	*TLR2*	0.816	*SPP1*	*VCAM1*	0.598	*TLR2*	*TIMP1*	0.439
FN1	*BGN*	0.807	*CYBB*	*TYROBP*	0.595	*MMP9*	*TYROBP*	0.438
ICAM1	*SELL*	0.804	*C1QB*	*PLEK*	0.593	*FN1*	*PLEK*	0.429
MMP9	*TLR2*	0.799	*C1QB*	*LCP2*	0.592	*LILRB2*	*TLR4*	0.429
BGN	*COL3A1*	0.798	*C1QB*	*VCAM1*	0.592	*CXCR4*	*LOX*	0.428
PECAM1	*ICAM1*	0.798	*LOX*	*TIMP1*	0.583	*PECAM1*	*CCR1*	0.426
PTPRC	*PLEK*	0.791	*MMP9*	*CCR1*	0.58	*C1QB*	*TLR2*	0.425
PECAM1	*CXCR4*	0.785	*FN1*	*TLR2*	0.579	*COL3A1*	*VCAM1*	0.425
CD163	*TLR2*	0.784	*MMP9*	*LOX*	0.579	*ICAM1*	*LOX*	0.421
TYROBP	*PLEK*	0.781	*PTPRC*	*C1QB*	0.579	*FN1*	*CCR1*	0.415
CXCR4	*MMP9*	0.78	*PTPRC*	*SPP1*	0.577	*BGN*	*IGF1*	0.411
TLR4	*CCR1*	0.772	*CXCL10*	*SELL*	0.576	*SPP1*	*COL3A1*	0.411
MMP9	*CXCL10*	0.769	*LILRB2*	*C1QB*	0.576	*CXCR4*	*LCP2*	0.41
PTPRC	*FN1*	0.768	*BGN*	*LOX*	0.575	*CXCL1*	*IGF1*	0.409
COL1A2	*LOX*	0.766	*PTPRC*	*IGF1*	0.575	*LILRB2*	*SELL*	0.403
CXCL1	*TLR4*	0.76	*CYBB*	*ICAM1*	0.57	*VCAM1*	*C3*	0.403
PECAM1	*FN1*	0.759	*PTPRC*	*MMP9*	0.57	*PTPRC*	*LOX*	0.401
CXCL1	*CAM1*	0.757	*BGN*	*TIMP1*	0.568			
FN1	*VCAM1*	0.75	*CCR1*	*TLR2*	0.568			

Table S5 Top 30 Nodes in WGCNA Network by Degree method

Gene Rank	Node	Rank Score	Fold Change	Gene Rank	Node	Rank Score	Fold Change
1	*CD53*	23	1.85	16	*LCP1*	15	2.13
1	*LILRB4*	23	4.61	17	*IFI16*	14	1.71
1	*SIGLEC7*	23	3.09	17	*RGS1*	14	1.83
4	*MS4A6A*	19	1.83	17	*SLCO2B1*	14	2.14
4	*MSR1*	19	6.02	17	*TYROBP*	14	2.04
6	*CCR1*	18	2.74	21	*C1QC*	13	2.08
7	*CYBB*	17	2.66	21	*CLEC7A*	13	2.52
7	*LAIR1*	17	2.48	21	*GBP1*	13	2.71
7	*SAMSN1*	17	2.19	21	*HCK*	13	2.05
10	*C1QB*	16	2.14	21	*PLA2G7*	13	7.61
10	*FPR3*	16	3.49	21	*TM6SF1*	13	1.63
10	*GPR65*	16	1.99	21	*TNFSF13B*	13	2.71
10	*HAVCR2*	16	4.21	28	*LCP2*	12	2.55
10	*IL2RA*	16	3.68	28	*SLA*	12	2.18
10	*PTPRC*	16	1.97	28	*TREM2*	12	11.85

Table S6 Edges in WGCNA Network by Degree method

Node1	Node2	Score	Node1	Node2	Score	Node1	Node2	Score
LCP1	*SLA*	0.475	*MS4A6A*	*LILRB4*	0.270	*LCP2*	*MS4A6A*	0.229
CD53	*LCP1*	0.373	*IL2RA*	*SIGLEC7*	0.270	*TNFSF13B*	*C1QB*	0.229
CD53	*SLA*	0.367	*C1QC*	*LILRB4*	0.270	*CD53*	*SAMSN1*	0.229
CD53	*PTPRC*	0.342	*C1QB*	*CYBB*	0.269	*TYROBP*	*LILRB4*	0.228
LCP1	*PTPRC*	0.338	*MS4A6A*	*CYBB*	0.269	*C1QB*	*PLA2G7*	0.228
C1QB	*HAVCR2*	0.333	*C1QC*	*TREM2*	0.269	*SLCO2B1*	*CYBB*	0.228
HAVCR2	*LILRB4*	0.330	*C1QB*	*LILRB4*	0.268	*SLCO2B1*	*HAVCR2*	0.228
C1QC	*LAIR1*	0.328	*LAIR1*	*CYBB*	0.268	*LCP2*	*PLA2G7*	0.227
C1QB	*LAIR1*	0.328	*IL2RA*	*LAIR1*	0.268	*TYROBP*	*CYBB*	0.227
C1QC	*HAVCR2*	0.325	*TNFSF13B*	*LAIR1*	0.268	*LCP2*	*CCR1*	0.227
C1QB	*SIGLEC7*	0.318	*MS4A6A*	*CCR1*	0.267	*LCP2*	*IL2RA*	0.225
PLA2G7	*HAVCR2*	0.318	*GPR65*	*CYBB*	0.267	*C1QC*	*PLA2G7*	0.224
C1QC	*SIGLEC7*	0.317	*CLEC7A*	*CYBB*	0.267	*HCK*	*CCR1*	0.223
PLA2G7	*FPR3*	0.314	*CYBB*	*MSR1*	0.265	*FPR3*	*TM6SF1*	0.223
TYROBP	*LAIR1*	0.312	*TREM2*	*HAVCR2*	0.265	*TNFSF13B*	*C1QC*	0.223
SIGLEC7	*LILRB4*	0.312	*PLA2G7*	*MS4A6A*	0.263	*CLEC7A*	*SIGLEC7*	0.222
FPR3	*CCR1*	0.312	*TYROBP*	*FPR3*	0.263	*IL2RA*	*C1QB*	0.221
C1QB	*C1QC*	0.311	*TNFSF13B*	*FPR3*	0.262	*LCP2*	*LILRB4*	0.220
SLA	*PTPRC*	0.310	*TNFSF13B*	*CYBB*	0.262	*HCK*	*C1QC*	0.220
LAIR1	*LILRB4*	0.309	*TREM2*	*MS4A6A*	0.262	*HCK*	*PLA2G7*	0.220
HAVCR2	*CCR1*	0.307	*C1QC*	*CYBB*	0.261	*CLEC7A*	*LAIR1*	0.220
SIGLEC7	*HAVCR2*	0.307	*LCP2*	*CYBB*	0.261	*HCK*	*C1QB*	0.219
FPR3	*MSR1*	0.306	*RGS1*	*CD53*	0.260	*CLEC7A*	*SAMSN1*	0.217
SIGLEC7	*FPR3*	0.304	*LCP2*	*FPR3*	0.260	*IL2RA*	*C1QC*	0.215
HAVCR2	*FPR3*	0.304	*LCP2*	*HAVCR2*	0.257	*RGS1*	*CYBB*	0.215
FPR3	*LILRB4*	0.304	*GPR65*	*FPR3*	0.257	*TM6SF1*	*MS4A6A*	0.215
IL2RA	*HAVCR2*	0.303	*PLA2G7*	*CCR1*	0.256	*LAIR1*	*TM6SF1*	0.214
TYROBP	*C1QC*	0.303	*C1QB*	*TREM2*	0.255	*HCK*	*MS4A6A*	0.214
SIGLEC7	*LAIR1*	0.302	*GPR65*	*LAIR1*	0.255	*RGS1*	*SLA*	0.214
FPR3	*MS4A6A*	0.298	*TNFSF13B*	*MS4A6A*	0.254	*CD53*	*GPR65*	0.211
LAIR1	*MS4A6A*	0.298	*LILRB4*	*CCR1*	0.253	*LCP2*	*GPR65*	0.211
PLA2G7	*CYBB*	0.298	*TREM2*	*FPR3*	0.252	*HCK*	*IL2RA*	0.211
TYROBP	*SIGLEC7*	0.298	*HCK*	*HAVCR2*	0.250	*IFI16*	*CYBB*	0.210
SIGLEC7	*CCR1*	0.297	*GPR65*	*MS4A6A*	0.249	*CLEC7A*	*MS4A6A*	0.209
SIGLEC7	*PLA2G7*	0.296	*SLCO2B1*	*FPR3*	0.248	*CLEC7A*	*CD53*	0.208
C1QB	*FPR3*	0.296	*CLEC7A*	*HAVCR2*	0.247	*PLA2G7*	*MSR1*	0.207
HAVCR2	*LAIR1*	0.295	*HAVCR2*	*GPR65*	0.247	*PTPRC*	*CYBB*	0.207
TYROBP	*C1QB*	0.294	*CLEC7A*	*FPR3*	0.246	*GBP1*	*CYBB*	0.207
TYROBP	*HAVCR2*	0.294	*LCP2*	*LAIR1*	0.246	*CLEC7A*	*PLA2G7*	0.207
HAVCR2	*MS4A6A*	0.293	*TYROBP*	*TREM2*	0.246	*TYROBP*	*CCR1*	0.206
C1QB	*MS4A6A*	0.292	*CYBB*	*SAMSN1*	0.246	*CLEC7A*	*LCP2*	0.205
C1QC	*FPR3*	0.290	*HCK*	*FPR3*	0.245	*TNFSF13B*	*LILRB4*	0.205
TREM2	*LAIR1*	0.290	*HCK*	*SIGLEC7*	0.245	*HAVCR2*	*SAMSN1*	0.205
C1QC	*MS4A6A*	0.289	*IL2RA*	*MS4A6A*	0.245	*TM6SF1*	*MSR1*	0.204
CYBB	*LILRB4*	0.288	*TNFSF13B*	*SIGLEC7*	0.244	*GPR65*	*PTPRC*	0.204
TNFSF13B	*HAVCR2*	0.287	*CD53*	*CYBB*	0.244	*TREM2*	*CYBB*	0.203
HAVCR2	*CYBB*	0.286	*SLCO2B1*	*LAIR1*	0.244	*LCP2*	*C1QB*	0.202

IL2RA	*FPR3*	0.285	*HCK*	*LAIR1*	0.243	*TYROBP*	*HCK*	0.202
HAVCR2	*MSR1*	0.285	*HCK*	*CYBB*	0.241	*LCP2*	*HCK*	0.202
LAIR1	*CCR1*	0.285	*LCP2*	*SIGLEC7*	0.241	*TNFSF13B*	*GPR65*	0.202
FPR3	*CYBB*	0.284	*IL2RA*	*CCR1*	0.239	*GPR65*	*MSR1*	0.202
SIGLEC7	*MS4A6A*	0.284	*C1QB*	*CCR1*	0.238	*CLEC7A*	*LCP1*	0.202
FPR3	*LAIR1*	0.284	*PLA2G7*	*LILRB4*	0.236	*FPR3*	*SAMSN1*	0.202
IL2RA	*CYBB*	0.283	*MSR1*	*CCR1*	0.236	*TREM2*	*LILRB4*	0.201
CYBB	*CCR1*	0.283	*HAVCR2*	*GBP1*	0.235	*LCP2*	*TNFSF13B*	0.201
SIGLEC7	*TREM2*	0.282	*RGS1*	*LCP1*	0.235	*IFI16*	*HAVCR2*	0.201
TYROBP	*MS4A6A*	0.279	*C1QC*	*CCR1*	0.234	*LCP2*	*MSR1*	0.201
SIGLEC7	*MSR1*	0.279	*IL2RA*	*LILRB4*	0.233	*IL2RA*	*PLA2G7*	0.201
PLA2G7	*LAIR1*	0.278	*HCK*	*LILRB4*	0.232	*TNFSF13B*	*CCR1*	0.201
LAIR1	*MSR1*	0.274	*SLCO2B1*	*MS4A6A*	0.231	*GPR65*	*CCR1*	0.201
SIGLEC7	*CYBB*	0.274	*SIGLEC7*	*GPR65*	0.231	*CLEC7A*	*HCK*	0.200
MS4A6A	*MSR1*	0.273	*SLCO2B1*	*SIGLEC7*	0.231	*SIGLEC7*	*TM6SF1*	0.200

Table S7 Biological functions and cancer relations of hub genes in PPI and WGCNA networks

Gene name	Gene ID*	Gene function Summary	Representative studies associated with cancer
**Common hub genes in PPI and WGCNA networks**			
TYROBP	7305	This gene encodes a transmembrane signaling polypeptide which contains an immunoreceptor tyrosine-based activation motif (ITAM) in its cytoplasmic domain. The encoded protein may associate with the killer-cell inhibitory receptor (KIR) family of membrane glycoproteins and may act as an activating signal transduction element.	Shabo, et al. found TYROBP overexpression was associated with skeletal and liver metastasis in patients with breast cancer[1].<br>Takamiya, et al. found TYROBP was mediated at the interaction between tumor associated macrophages and cancer cells and contributed to tumor progression in lung cancer[2].
C1QB	713	This gene encodes the B-chain polypeptide of serum complement subcomponent C1q, which associates with C1r and C1s to yield the first component of the serum complement system. C1q is composed of 18 polypeptide chains which include 6 A-chains, 6 B-chains, and 6 C-chains.	Luo, et al. found altered expression of C1QB in blood could be identified as a biomarker for early melanoma[3].
PTPRC	5788	The protein encoded by this gene is a member of the protein tyrosine phosphatase (PTP) family. PTPs are known to be signaling molecules that regulate a variety of cellular processes including cell growth, differentiation, mitosis, and oncogenic transformation.	Ju, et al. reported that PTPRC polymorphism was associated with susceptibility to diffuse-type gastric cancer and gene expression [4].<br>Ahmadvand, et al. found PTPRC^{+} TILs served as a negative predictor for OS and PFS of breast cancer patients[5].
LCP2	3937	This gene encodes an adapter protein that acts as a substrate of the T cell antigen receptor (TCR)-activated protein tyrosine kinase pathway. The encoded protein associates with growth factor receptor bound protein 2, and is thought to play a role TCR-mediated intracellular signal transduction.	Chu, et al identified LCP2 as one of the DEGs which contributed to liver metastasis of colon cancer[6].
CYBB	1536	Cytochrome b (-245) is composed of cytochrome b alpha (CYBA) and beta (CYBB) chain. It has been proposed as a primary component of the microbicidal oxidase system of phagocytes. CYBB deficiency is one of five described biochemical defects associated with chronic granulomatous disease (CGD).	Wang, et al identified that CYBB as a new biomarker and a potential therapeutic target for gastric cancer [7].<br>Zhang, et al. identified CYBB as one of the hub genes in hypoxia networks in ovarian cancer via bioinformatics analysis[8].
CCR1	1230	This gene encodes a member of the beta chemokine receptor family, which is predicted to be a seven transmembrane protein similar to G protein-coupled receptors. The ligands of this receptor include macrophage inflammatory protein 1 alpha (MIP-1 alpha), regulated on activation normal T expressed and secreted protein (RANTES), monocyte chemoattractant protein 3 (MCP-3), and myeloid progenitor inhibitory factor-1 (MPIF-1).	Itatani, et al. found that CCR1+ myeloid cells promoted tumor invasion and liver metastasis in colorectal cancer [9].<br>Sugasawa, et al found that gastric cancer cells showed positive expression of CCR1 [10].
**Other hub genes in PPI network**			
LOX	4015	This gene encodes a member of the lysyl oxidase family of proteins. Alternative splicing results in multiple transcript variants, at least one of which encodes a preproprotein that is proteolytically processed to generate a regulatory propeptide and the mature enzyme.	Zhao, et al reported that LOX inhibition downregulated MMP-2 and MMP-9 in gastric cancer tissues and cells [11].
COL3A1	1281	This gene encodes the pro-alpha1 chains of type III collagen, a fibrillar collagen that is	Liu, et al. identified COL3A1 as one of the hub

		found in extensible connective tissues such as skin, lung, uterus, intestine and the vascular system, frequently in association with type I collagen.	genes correlated with the pathogenesis of gastric cancer based on bioinformatics analysis[12].
FN1	2335	This gene encodes fibronectin, a glycoprotein present in a soluble dimeric form in plasma, and in a dimeric or multimeric form at the cell surface and in extracellular matrix. The encoded preproprotein is proteolytically processed to generate the mature protein.	Wang, et al. found that microRNA-432 targeting FN1 inhibited cell proliferation and invasion of cervical cancer[13].
FBN1	2200	This gene encodes a member of the fibrillin family of proteins. The encoded preproprotein is proteolytically processed to generate two proteins including the extracellular matrix component fibrillin-1 and the protein hormone asprosin. Fibrillin-1 is an extracellular matrix glycoprotein that serves as a structural component of calcium-binding microfibrils.	Shi, et al. found that FBN1 as a potential biomarker of progression and prognosis in bladder cancer[14]. Yang, et al reported that MiR-133b inhibited the proliferative, migratory and invasive abilities of GC cells by increasing FBN1 expression [15].
CXCR4	7852	This gene encodes a CXC chemokine receptor specific for stromal cell-derived factor-1. The protein has 7 transmembrane regions and is located on the cell surface. It acts with the CD4 protein to support HIV entry into cells and is also highly expressed in breast cancer cells.	Lzumi, et al. found that the inhibition of CXCL12/CXCR4 signaling in GC cells may be a promising therapeutic strategy against GC cell invasion [16].
PECAM1	5175	The protein encoded by this gene is found on the surface of platelets, monocytes, neutrophils, and some types of T-cells, and makes up a large portion of endothelial cell intercellular junctions. The encoded protein is a member of the immunoglobulin superfamily and is likely involved in leukocyte migration, angiogenesis, and integrin activation.	Terashima, et al. reported that PECAM1 was associated with hematogenous, lymph Node, and peritoneal recurrence in stage II/III gastric cancer patients enrolled in the ACTS-GC Study[17].
BGN	633	This gene encodes a member of the small leucine-rich proteoglycan (SLRP) family of proteins. The encoded preproprotein is proteolytically processed to generate the mature protein, which plays a role in bone growth, muscle development and regeneration, and collagen fibril assembly in multiple tissues.	Sun, et al. identified BGN as one of the core genes associated with tumor metastasis in gastric cancer[18].
IGF1	3479	The protein encoded by this gene is similar to insulin in function and structure and is a member of a family of proteins involved in mediating growth and development. The encoded protein is processed from a precursor, bound by a specific receptor, and secreted. Defects in this gene are a cause of insulin-like growth factor I deficiency.	Du, et al found that IGF1 was upregulated to promote gastric cancer metastasis [19].
TLR2	7097	The protein encoded by this gene is a member of the Toll-like receptor (TLR) family which plays a fundamental role in pathogen recognition and activation of innate immunity. This protein is a cell-surface protein that can form heterodimers with other TLR family members to recognize conserved molecules derived from microorganisms known as pathogen-associated molecular patterns (PAMPs).	West, et al. identified a TLR2-regulated gene signature as a predictor of poor outcomes in patients with gastric cancer [20].
COL1A2	1278	This gene encodes the pro-alpha2 chain of type I collagen whose triple helix comprises two alpha1 chains and one alpha2 chain. Type I is a fibril-forming collagen found in most connective tissues and is abundant in bone, cornea, dermis and tendon.	Li, et al. identified COL1A2 as a candidate prognostic factor in gastric cancer[21]. Ao, et al. found that silencing of COL1A2 inhibited gastric cancer cell proliferation, migration and invasion[22].
VCAM1	7412	This gene is a member of the Ig superfamily and encodes a cell surface sialoglycoprotein expressed by cytokine-activated endothelium. This type I membrane protein mediates leukocyte-endothelial cell adhesion and signal transduction, and may play a role in the development of artherosclerosis and rheumatoid arthritis.	Shen, et al. found that cancer-associated fibroblasts-derived VCAM1 facilitated tumor invasion in gastric cancer [23].
COL1A1	1277	This gene encodes the pro-alpha1 chains of type I collagen whose triple helix comprises two alpha1 chains and one alpha2 chain. Type I is a fibril-forming collagen found in most	Liu, et al. identified COL1A1 as one of the genes in prognostic signature of gastric cancer [24].

		connective tissues and is abundant in bone, cornea, dermis and tendon.	Wang, et al. found that miR-129-5p suppresses gastric cancer cell invasion by inhibiting COL1A1[25].
SELL	6402	This gene encodes a cell surface adhesion molecule that belongs to a family of adhesion/homing receptors. The encoded protein contains a C-type lectin-like domain, a calcium-binding epidermal growth factor-like domain, and two short complement-like repeats. The gene product is required for binding and subsequent rolling of leucocytes on endothelial cells, facilitating their migration into secondary lymphoid organs and inflammation sites.	Few studies were reported
C3	718	Its activation is required for both classical and alternative complement activation pathways. The encoded preproprotein is proteolytically processed to generate alpha and beta subunits that form the mature protein, which is then further processed to generate numerous peptide products.	Boire, et al. found that C3 adapted the cerebrospinal fluid for leptomeningeal metastasis[26].
TLR4	7099	The protein encoded by this gene is a member of the Toll-like receptor (TLR) family which plays a fundamental role in pathogen recognition and activation of innate immunity. TLRs are highly conserved from Drosophila to humans and share structural and functional similarities. They recognize pathogen-associated molecular patterns that are expressed on infectious agents, and mediate the production of cytokines necessary for the development of effective immunity.	Zandi, et al. found that TLR4 blockade suppressed ovarian and breast cancer cells invasion through inhibition of extracellular matrix degradation and EMT[27].
LILRB2	10288	This gene is a member of the leukocyte immunoglobulin-like receptor (LIR) family, which is found in a gene cluster at chromosomal region 19q13.4. The encoded protein belongs to the subfamily B class of LIR receptors which contain two or four extracellular immunoglobulin domains, a transmembrane domain, and two to four cytoplasmic immunoreceptor tyrosine-based inhibitory motifs (ITIMs)	Shao, et al. found that LILRB2 was critical for endometrial cancer progression[28].<br>Liu, et al. found that LILRB2 promoted the propagation of lung cancer cells[29].
ICAM1	3383	This gene encodes a cell surface glycoprotein which is typically expressed on endothelial cells and cells of the immune system. It binds to integrins of type CD11a / CD18, or CD11b / CD18 and is also exploited by Rhinovirus as a receptor.	Li, et al identified LILRB2 as one of the genes in metastasis model of human gastric cancer in nude mice [30].
TIMP1	7076	This gene belongs to the TIMP gene family. The proteins encoded by this gene family are natural inhibitors of the matrix metalloproteinases (MMPs), a group of peptidases involved in degradation of the extracellular matrix.	Liu, et al. identified TIMP1 as one of the key genes associated with the pathogenesis and prognosis of gastric cancer by bioinformatic analysis [31].
SPP1	6696	The protein encoded by this gene is involved in the attachment of osteoclasts to the mineralized bone matrix. The encoded protein is secreted and binds hydroxyapatite with high affinity.	Choe, et al. found that upregulation of SPP1 was related to poor survival outcomes in colorectal cancer[32].
CD163	9332	The protein encoded by this gene is a member of the scavenger receptor cysteine-rich (SRCR) superfamily, and is exclusively expressed in monocytes and macrophages. It functions as an acute phase-regulated receptor involved in the clearance and endocytosis of hemoglobin/haptoglobin complexes by macrophages, and may thereby protect tissues from free hemoglobin-mediated oxidative damage.	Ding, et al. identified CD163 as a novel biomarker for colorectal cancer[33].<br>Shiraishi, et al. found that CD163 was required for protumoral activation of macrophages in human and murine sarcoma[34].
MMP9	4318	Proteins of the matrix metalloproteinase (MMP) family are involved in the breakdown of extracellular matrix in normal physiological processes, such as embryonic development, reproduction, and tissue remodeling, as well as in disease processes, such as arthritis and metastasis.	Ren, et al. found that coronin3 promoted gastric cancer metastasis via the up-regulation of MMP9 and cathepsin K [35].
**CXCL10**	3627	This antimicrobial gene encodes a chemokine of the CXC subfamily and ligand for the	Zhou, et al reported that CXCL10/CXCR3 axis

		receptor CXCR3. Binding of this protein to CXCR3 results in pleiotropic effects, including stimulation of monocytes, natural killer and T-cell migration, and modulation of adhesion molecule expression.	promoted the invasion of gastric cancer [36].
PLEK	5341	PLEK (Pleckstrin) is a Protein Coding gene. Diseases associated with PLEK include Aarskog-Scott Syndrome and Agammaglobulinemia, X-Linked. Among its related pathways are Response to elevated platelet cytosolic Ca^{2+}.	Few studies were reported
CXCL1	2919	This antimicrobial gene encodes a member of the CXC subfamily of chemokines. The encoded protein is a secreted growth factor that signals through the G-protein coupled receptor, CXC receptor 2. This protein plays a role in inflammation and as a chemoattractant for neutrophils.	Cheng, et al. found that overexpression of CXCL1 and its receptor CXCR2 promoted tumor invasion in gastric cancer [37].
**Other hub genes in WGCNA network**			
RGS1	5996	This gene encodes a member of the regulator of G-protein signalling family. This protein is located on the cytosolic side of the plasma membrane and contains a conserved, 120 amino acid motif called the RGS domain. The protein attenuates the signalling activity of G-proteins by binding to activated, GTP-bound G alpha subunits and acting as a GTPase activating protein (GAP), increasing the rate of conversion of the GTP to GDP.	Tanabe, et al. found that RGS1 was up-regulated in diffuse-type GC cells[38].
CLEC7A	64581	This gene encodes a member of the C-type lectin/C-type lectin-like domain (CTL/CTLD) superfamily. The encoded glycoprotein is a small type II membrane receptor with an extracellular C-type lectin-like domain fold and a cytoplasmic domain with an immunoreceptor tyrosine-based activation motif. It functions as a pattern-recognition receptor that recognizes a variety of beta-1,3-linked and beta-1,6-linked glucans from fungi and plants, and in this way plays a role in innate immune response.	Few studies were reported
LILRB4	11006	This gene is a member of the leukocyte immunoglobulin-like receptor (LIR) family, which is found in a gene cluster at chromosomal region 19q13.4. The encoded protein belongs to the subfamily B class of LIR receptors which contain two or four extracellular immunoglobulin domains, a transmembrane domain, and two to four cytoplasmic immunoreceptor tyrosine-based inhibitory motifs (ITIMs).	Zhang, et al. reported that LILRB4 played a key role in gastric cancer immune escape [39].
HCK	3055	The protein encoded by this gene is a member of the Src family of tyrosine kinases. This protein is primarily hemopoietic, particularly in cells of the myeloid and B-lymphoid lineages. It may help couple the Fc receptor to the activation of the respiratory burst. In addition, it may play a role in neutrophil migration and in the degranulation of neutrophils.	Poh, et al. found that HCK inhibition impaired STAT3-dependent gastric tumor growth in mice[40].
SLCO2B1	11309	This locus encodes a member of the organic anion-transporting polypeptide family of membrane proteins. The protein encoded by this locus may function in regulation of placental uptake of sulfated steroids.	Hahn,et al. found that SLCO2B1 might contribute to castration resistance of prostate cancer[41].
C1QC	714	This gene encodes the C-chain polypeptide of serum complement subcomponent C1q, which associates with C1r and C1s to yield the first component of the serum complement system.	Few studies were reported
GBP1	2633	Guanylate binding protein expression is induced by interferon. Guanylate binding proteins are characterized by their ability to specifically bind guanine nucleotides (GMP, GDP, and GTP) and are distinguished from the GTP-binding proteins by the presence of 2 binding motifs rather than 3.	Ji, et al. reported that overexpression of GBP1 predicted poor prognosis and promotes tumor growth in human glioblastoma multiforme[42].
LAIR1	3903	The protein encoded by this gene is an inhibitory receptor found on peripheral	Jingushi, et al. found that LAIR1 promoted

		mononuclear cells, including natural killer cells, T cells, and B cells. Inhibitory receptors regulate the immune response to prevent lysis of cells recognized as self. The gene is a member of both the immunoglobulin superfamily and the leukocyte-associated inhibitory receptor family.	tumorigenesis in renal cell carcinoma [43].
HAVCR2	84868	The protein encoded by this gene belongs to the immunoglobulin superfamily, and TIM family of proteins. CD4-positive T helper lymphocytes can be divided into types 1 (Th1) and 2 (Th2) on the basis of their cytokine secretion patterns.	Wang, et al found that HAVCR2 expression were independent prognostic factor for patients with gastric cancer [44].
FPR3	2359	FPR3 (Formyl Peptide Receptor 3) is a Protein Coding gene. Diseases associated with FPR3 include Rubeosis Iridis. Among its related pathways are Signaling by GPCR and Peptide ligand-binding receptors.	Cheng, et al reported that FPR3 were associated with invasiveness of gastric cancer [45].
TNFSF13B	10673	The protein encoded by this gene is a cytokine that belongs to the tumor necrosis factor (TNF) ligand family. This cytokine is a ligand for receptors TNFRSF13B/TACI, TNFRSF17/BCMA, and TNFRSF13C/BAFFR.	Li, et al identified TNFSF13B as one of the hub genes in kidney renal clear cell carcinoma microenvironment [46].
TREM2	54209	This gene encodes a membrane protein that forms a receptor signaling complex with the TYRO protein tyrosine kinase binding protein. The encoded protein functions in immune response and may be involved in chronic inflammation by triggering the production of constitutive inflammatory cytokines.	Zhang, et al found that high TREM2 expression correlates with poor prognosis in gastric cancer [47]. Tang, et al reported that TREM2 acts as a tumor suppressor in hepatocellular carcinoma [48].
PLA2G7	7941	The protein encoded by this gene is a secreted enzyme that catalyzes the degradation of platelet-activating factor to biologically inactive products. Defects in this gene are a cause of platelet-activating factor acetylhydrolase deficiency.	Lehtinen, et al found that PLA2G7 regulated EMT in cultured breast cancer cells [49].
SIGLEC7	27036	SIGLEC7 (Sialic Acid Binding Ig Like Lectin 7) is a Protein Coding gene. Diseases associated with SIGLEC7 include Congenital Disorder Of Glycosylation, Type Iic and Autoimmune Disease Of Peripheral Nervous System.	Few studies were reported
TM6SF1	53346	TM6SF1 (Transmembrane 6 Superfamily Member 1) is a Protein Coding gene.	Few studies were reported
IFI16	3428	This gene encodes a member of the HIN-200 (hematopoietic interferon-inducible nuclear antigens with 200 amino acid repeats) family of cytokines. The encoded protein contains domains involved in DNA binding, transcriptional regulation, and protein-protein interactions.	Yang, et al found that IFI16 showed lower mRNA expression in the colorectal tumor tissues [50].
MS4A6A	64231	This gene encodes a member of the membrane-spanning 4A gene family. Members of this nascent protein family are characterized by common structural features and similar intron/exon splice boundaries and display unique expression patterns among hematopoietic cells and nonlymphoid tissues.	Pan, et al reported that MS4A6A was relevant to pathological grade and prognosis in ovarian cancer [51].
CD53	963	The protein encoded by this gene is a member of the transmembrane 4 superfamily, also known as the tetraspanin family. Most of these members are cell-surface proteins that are characterized by the presence of four hydrophobic domains.	Few studies were reported
MSR1	4481	This gene encodes the class A macrophage scavenger receptors, which include three different types (1, 2, 3) generated by alternative splicing of this gene. These receptors or isoforms are macrophage-specific trimeric integral membrane glycoproteins and have been implicated in many macrophage-associated physiological and pathological processes including atherosclerosis, Alzheimer's disease, and host defense.	Rose, et al found that MSR1 repeats modulated gene expression and affected risk of breast and prostate cancer [52].
SAMSN1	64092	SAMSN1 is a member of a novel gene family of putative adaptors and scaffold proteins containing SH3 and SAM (sterile alpha motif) domains	Kanda, et al reported that downregulation of SAMSN1 transcription affected the progression and recurrence of gastric cancer [53].

			Sueoka, et al found that suppression of SAMSN1 expression is associated with the malignant phenotype of hepatocellular carcinoma [54].
LCP1	3936	Plastins are a family of actin-binding proteins that are conserved throughout eukaryote evolution and expressed in most tissues of higher eukaryotes. In humans, two ubiquitous plastin isoforms (L and T) have been identified.	Koide, et al reported that LCP1 is a useful biomarker for determining progression of oral squamous cell carcinomas [55].
GPR65	8477	GPR65 (G Protein-Coupled Receptor 65) is a Protein Coding gene. Diseases associated with GPR65 include Gastric Cancer and B-Cell Childhood Acute Lymphoblastic Leukemia. Among its related pathways are Signaling by GPCR and RET signaling.	Li, et al reported that Long non-coding RNA GPR65-1 was up-regulated in gastric cancer and promoted tumor growth [56].
SLA	6503	SLA (Src Like Adaptor) is a Protein Coding gene. Diseases associated with SLA include Inflammatory Bowel Disease 25.	Few studies were reported
IL2RA	3559	The interleukin 2 (IL2) receptor alpha (IL2RA) and beta (IL2RB) chains, together with the common gamma chain (IL2RG), constitute the high-affinity IL2 receptor.	Jia, et al found that IL2RA polymorphisms were associated with lung cancer risk in the Chinese Han population [57].

*derived from: https://www.ncbi.nlm.nih.gov/gene;

Note: all information was achieved from GENE (https://www.ncbi.nlm.nih.gov/gene) and GeneCards (http://www.genecards.org)

References

1. Shabo I, Olsson H, Stål O, Svanvik J. Breast cancer expression of DAP12 is associated with skeletal and liver metastases and poor survival. Clin Breast Cancer. 2013;13(5):371-377.

2. Takamiya R, Ohtsubo K, Takamatsu S, Taniguchi N, Angata T. The interaction between Siglec-15 and tumor-associated sialyl-Tn antigen enhances TGF-β secretion from monocytes/macrophages through the DAP12-Syk pathway. Glycobiology. 2013;23(2):178-187.

3. Luo Y, Robinson S, Fujita J, et al. Transcriptome profiling of whole blood cells identifies PLEK2 and C1QB in human melanoma. PLoS One. 2011;6(6):e20971.

4. Camacho M, Agüero A, Sumarroca A, et al. Prognostic value of CD45 transcriptional expression in head and neck cancer. Eur Arch Otorhinolaryngol. 2018;275(1):225-232.

5. Ahmadvand S, Faghih Z, Montazer M, et al. Importance of CD45RO+ tumor-infiltrating lymphocytes in post-operative survival of breast cancer patients. Cell Oncol (Dordr). 2019;42(3):343-356.

6. Chu S, Wang H, Yu M. A putative molecular network associated with colon cancer metastasis constructed from microarray data. World J Surg Oncol. 2017;15(1):115.

7. Wang P, Shi Q, Deng WH, et al. Relationship between expression of NADPH oxidase 2 and invasion and prognosis of human gastric cancer. World J Gastroenterol. 2015;21(20):6271-6279. doi:10.3748/wjg.v21.i20.6271

8. Zhang K, Kong X, Feng G, et al. Investigation of hypoxia networks in ovarian cancer via bioinformatics analysis. J Ovarian Res. 2018;11(1):16. Published 2018 Feb 26.

9. Itatani Y, Kawada K, Fujishita T, et al. Loss of SMAD4 from colorectal cancer cells promotes CCL15 expression to recruit CCR1+ myeloid cells and facilitate liver metastasis. Gastroenterology. 2013;145(5):1064-1075.e11.

10. Sugasawa H, Ichikura T, Tsujimoto H, et al. Prognostic significance of expression of CCL5/RANTES receptors in patients with gastric cancer. J Surg Oncol. 2008;97(5):445-450. doi:10.1002/jso.20984

11. Zhao L, Niu H, Liu Y, et al. LOX inhibition downregulates MMP-2 and MMP-9 in gastric cancer tissues and cells. J Cancer. 2019;10(26):6481-6490. Published 2019 Oct 20. doi:10.7150/jca.33223

12. Liu X, Wu J, Zhang D, et al. Identification of Potential Key Genes Associated With the Pathogenesis and Prognosis of Gastric Cancer Based on Integrated Bioinformatics Analysis. Front Genet. 2018;9:265.
13. Wang S, Gao B, Yang H, Liu X, Wu X, Wang W. MicroRNA-432 is downregulated in cervical cancer and directly targets FN1 to inhibit cell proliferation and invasion. Oncol Lett. 2019;18(2):1475-1482.
14. Shi S, Tian B. Identification of biomarkers associated with progression and prognosis in bladder cancer via co-expression analysis. Cancer Biomark. 2019;24(2):183-193.
15. Yang D, Zhao D, Chen X. MiR-133b inhibits proliferation and invasion of gastric cancer cells by up-regulating FBN1 expression. Cancer Biomark. 2017;19(4):425-436. doi:10.3233/CBM-160421
16. Martinez-Ordoñez A, Seoane S, Cabezas P, et al. Breast cancer metastasis to liver and lung is facilitated by Pit-1-CXCL12-CXCR4 axis. Oncogene. 2018;37(11):1430-1444.
17. Terashima M, Ichikawa W, Ochiai A, et al. TOP2A, GGH, and PECAM1 are associated with hematogenous, lymph node, and peritoneal recurrence in stage II/III gastric cancer patients enrolled in the ACTS-GC study. Oncotarget. 2017;8(34):57574-57582.
18. Sun C, Yuan Q, Wu D, Meng X, Wang B. Identification of core genes and outcome in gastric cancer using bioinformatics analysis. Oncotarget. 2017;8(41):70271-70280.
19. Du F, Feng W, Chen S, et al. Sex determining region Y-box 12 (SOX12) promotes gastric cancer metastasis by upregulating MMP7 and IGF1. Cancer Lett. 2019;452:103-118. doi:10.1016/j.canlet.2019.03.035
20. West AC, Tang K, Tye H, et al. Identification of a TLR2-regulated gene signature associated with tumor cell growth in gastric cancer. Oncogene. 2017;36(36):5134-5144. doi:10.1038/onc.2017.121
21. Li J, Ding Y, Li A. Identification of COL1A1 and COL1A2 as candidate prognostic factors in gastric cancer. World J Surg Oncol. 2016;14(1):297.
22. Ao R, Guan L, Wang Y, Wang JN. Silencing of COL1A2, COL6A3, and THBS2 inhibits gastric cancer cell proliferation, migration, and invasion while promoting apoptosis through the PI3k-Akt signaling pathway. J Cell Biochem. 2018;119(6):4420-4434.
23. Shen J, Zhai J, You Q, et al. Cancer-associated fibroblasts-derived VCAM1 induced by H. pylori infection facilitates tumor invasion in gastric cancer. Oncogene. 2020;39(14):2961-2974. doi:10.1038/s41388-020-1197-4
24. Liu X, Wu J, Zhang D, et al. Identification of Potential Key Genes Associated With the Pathogenesis and Prognosis of Gastric Cancer Based on Integrated Bioinformatics Analysis. Front Genet. 2018;9:265. Published 2018 Jul 17. doi:10.3389/fgene.2018.00265
25. Wang Q, Yu J. MiR-129-5p suppresses gastric cancer cell invasion and proliferation by inhibiting COL1A1. Biochem Cell Biol. 2018;96(1):19-25.
26. Boire A, Zou Y, Shieh J, Macalinao DG, Pentsova E, Massagué J. Complement Component 3 Adapts the Cerebrospinal Fluid for Leptomeningeal Metastasis. Cell. 2017;168(6):1101-1113.e13.
27. Zandi Z, Kashani B, Poursani EM, et al. TLR4 blockade using TAK-242 suppresses ovarian and breast cancer cells invasion through the inhibition of extracellular matrix degradation and epithelial-mesenchymal transition. Eur J Pharmacol. 2019;853:256-263.
28. Shao H, Ma L, Jin F, Zhou Y, Tao M, Teng Y. Immune inhibitory receptor LILRB2 is critical for the endometrial cancer progression. Biochem Biophys Res Commun. 2018;506(1):243-250.
29. Liu X, Yu X, Xie J, et al. ANGPTL2/LILRB2 signaling promotes the propagation of lung cancer cells. Oncotarget. 2015;6(25):21004-21015.
30. Li K, Du H, Lian X, et al. Establishment and characterization of a metastasis model of human gastric cancer in nude mice. BMC Cancer. 2016;16:54. Published 2016 Feb 3. doi:10.1186/s12885-016-2101-z
31. Liu X, Wu J, Zhang D, et al. Identification of Potential Key Genes Associated With the Pathogenesis and Prognosis of Gastric Cancer Based on Integrated Bioinformatics

Analysis. Front Genet. 2018;9:265. Published 2018 Jul 17. doi:10.3389/fgene.2018.00265
32. Choe EK, Yi JW, Chai YJ, Park KJ. Upregulation of the adipokine genes ADIPOR1 and SPP1 is related to poor survival outcomes in colorectal cancer. J Surg Oncol. 2018;117(8):1833-1840.
33. Ding D, Yao Y, Yang C, Zhang S. Identification of mannose receptor and CD163 as novel biomarkers for colorectal cancer. Cancer Biomark. 2018;21(3):689-700.
34. Shiraishi D, Fujiwara Y, Horlad H, et al. CD163 Is Required for Protumoral Activation of Macrophages in Human and Murine Sarcoma. Cancer Res. 2018;78(12):3255-3266.
35. Ren G, Tian Q, An Y, et al. Coronin 3 promotes gastric cancer metastasis via the up-regulation of MMP-9 and cathepsin K. Mol Cancer. 2012;11:67. Published 2012 Sep 14. doi:10.1186/1476-4598-11-67
36. Zhou H, Wu J, Wang T, Zhang X, Liu D. CXCL10/CXCR3 axis promotes the invasion of gastric cancer via PI3K/AKT pathway-dependent MMPs production. Biomed Pharmacother. 2016;82:479-488. doi:10.1016/j.biopha.2016.04.069
37. Cheng WL, Wang CS, Huang YH, Tsai MM, Liang Y, Lin KH. Overexpression of CXCL1 and its receptor CXCR2 promote tumor invasion in gastric cancer [published correction appears in Ann Oncol. 2011 Nov;22(11):2537]. Ann Oncol. 2011;22(10):2267-2276. doi:10.1093/annonc/mdq739
38. Tanabe S, Aoyagi K, Yokozaki H, Sasaki H. Regulated genes in mesenchymal stem cells and gastric cancer. World J Stem Cells. 2015;7(1):208-222.
39. Zhang Y, Lu N, Xue Y, et al. Expression of immunoglobulin-like transcript (ILT)2 and ILT3 in human gastric cancer and its clinical significance. Mol Med Rep. 2012;5(4):910-916. doi:10.3892/mmr.2012.744
40. Poh AR, Dwyer AR, Eissmann MF, et al. Inhibition of the SRC Kinase HCK Impairs STAT3-Dependent Gastric Tumor Growth in Mice. Cancer Immunol Res. 2020;8(4):428-435. doi:10.1158/2326-6066.CIR-19-0623
41. Fujimoto N, Kubo T, Inatomi H, et al. Polymorphisms of the androgen transporting gene SLCO2B1 may influence the castration resistance of prostate cancer and the racial differences in response to androgen deprivation. Prostate Cancer Prostatic Dis. 2013;16(4):336-340.
42. Ji X, Zhu H, Dai X, et al. Overexpression of GBP1 predicts poor prognosis and promotes tumor growth in human glioblastoma multiforme. Cancer Biomark. 2019;25(3):275-290.
43. Jingushi K, Uemura M, Nakano K, et al. Leukocyte-associated immunoglobulin-like receptor 1 promotes tumorigenesis in RCC. Oncol Rep. 2019;41(2):1293-1303. doi:10.3892/or.2018.6875.
44. Wang Y, Zhao E, Zhang Z, Zhao G, Cao H. Association between Tim-3 and Gal-9 expression and gastric cancer prognosis. Oncol Rep. 2018;40(4):2115-2126. doi:10.3892/or.2018.6627.
45. Cheng TY, Wu MS, Lin JT, et al. Annexin A1 is associated with gastric cancer survival and promotes gastric cancer cell invasiveness through the formyl peptide receptor/extracellular signal-regulated kinase/integrin beta-1-binding protein 1 pathway. Cancer. 2012;118(23):5757-5767. doi:10.1002/cncr.27565.
46. Li S, Xu W. Mining TCGA database for screening and identification of hub genes in kidney renal clear cell carcinoma microenvironment [published online ahead of print, 2019 Nov 7]. J Cell Biochem. 2019;10.1002/jcb.29511. doi:10.1002/jcb.29511
47. Zhang X, Wang W, Li P, Wang X, Ni K. High TREM2 expression correlates with poor prognosis in gastric cancer. Hum Pathol. 2018;72:91-99. doi:10.1016/j.humpath.2017.10.026
48. Tang W, Lv B, Yang B, et al. TREM2 acts as a tumor suppressor in hepatocellular carcinoma by targeting the PI3K/Akt/β-catenin pathway. Oncogenesis. 2019;8(2):9. Published 2019 Jan 25. doi:10.1038/s41389-018-0115-x.
49. Lehtinen L, Vainio P, Wikman H, et al. PLA2G7 associates with hormone receptor negativity in clinical breast cancer samples and regulates epithelial-mesenchymal transition

in cultured breast cancer cells. J Pathol Clin Res. 2017;3(2):123-138. Published 2017 Apr 4. doi:10.1002/cjp2.69
50. Yang CA, Huang HY, Chang YS, Lin CL, Lai IL, Chang JG. DNA-Sensing and Nuclease Gene Expressions as Markers for Colorectal Cancer Progression. Oncology. 2017;92(2):115-124. doi:10.1159/000452281
51. Pan X, Chen Y, Gao S. Four genes relevant to pathological grade and prognosis in ovarian cancer [published online ahead of print, 2020 May 13]. Cancer Biomark. 2020;10.3233/CBM-191162. doi:10.3233/CBM-191162
52. Rose AM, Krishan A, Chakarova CF, et al. MSR1 repeats modulate gene expression and affect risk of breast and prostate cancer. Ann Oncol. 2018;29(5):1292-1303. doi:10.1093/annonc/mdy082
53. Kanda M, Shimizu D, Sueoka S, et al. Prognostic relevance of SAMSN1 expression in gastric cancer. Oncol Lett. 2016;12(6):4708-4716. doi:10.3892/ol.2016.5233
54. Sueoka S, Kanda M, Sugimoto H, et al. Suppression of SAMSN1 Expression is Associated with the Malignant Phenotype of Hepatocellular Carcinoma. Ann Surg Oncol. 2015;22 Suppl 3:S1453-S1460. doi:10.1245/s10434-015-4524-1
55. Koide N, Kasamatsu A, Endo-Sakamoto Y, et al. Evidence for Critical Role of Lymphocyte Cytosolic Protein 1 in Oral Cancer. Sci Rep. 2017;7:43379. Published 2017 Feb 23. doi:10.1038/srep43379
56. Li Y, Shen Z, Wang B, et al. Long non-coding RNA GPR65-1 is up-regulated in gastric cancer and promotes tumor growth through the PTEN-AKT-slug signaling pathway. Cell Cycle. 2018;17(6):759-765. doi:10.1080/15384101.2018.1426414
57. Jia Z, Zhang Z, Yang Q, Deng C, Li D, Ren L. Effect of IL2RA and IL2RB gene polymorphisms on lung cancer risk. Int Immunopharmacol. 2019;74:105716. doi:10.1016/j.intimp.2019.105716

Table S8 Association of hub genes in PPI and WGCNA networks with overall survival in TCGA-STAD

	mRNA expression		HR	95% CI		*P*
	Low	High		Bottom	Top	
Common hub genes in PPI and WGCNA network						
TYROBP	160	207	1.456	1.038	2.042	0.029
C1QB	146	221	1.475	1.037	2.098	0.030
PTPRC	256	111	1.301	0.923	1.832	0.132
LCP2	217	150	1.272	0.917	1.764	0.148
CYBB	113	254	1.287	0.882	1.877	0.189
CCR1	226	141	1.148	0.825	1.598	0.412
Other hub genes in PPI network						
LOX	221	146	1.716	1.234	2.385	0.001
COL3A1	270	97	1.739	1.233	2.454	0.001
FN1	249	118	1.706	1.224	2.377	0.001
FBN1	262	105	1.687	1.204	2.365	0.002
CXCR4	207	160	1.649	1.190	2.287	0.002
PECAM1	275	92	1.613	1.136	2.289	0.007
BGN	103	264	1.716	1.141	2.580	0.009
IGF1	246	121	1.486	1.066	2.072	0.019
TLR2	97	270	1.645	1.060	2.553	0.025
COL1A2	268	99	1.483	1.043	2.109	0.027
VCAM1	117	250	1.527	1.031	2.263	0.033
COL1A1	93	274	1.531	1.006	2.328	0.045
SELL	100	267	1.499	0.997	2.253	0.050
C3	256	111	1.397	0.997	1.957	0.051
TLR4	242	125	1.372	0.985	1.912	0.060
LILRB2	135	232	1.394	0.975	1.993	0.067
ICAM1	152	215	1.365	0.968	1.927	0.075
TIMP1	275	92	1.361	0.954	1.942	0.087
SPP1	201	166	1.279	0.922	1.774	0.140
CD163	263	104	1.235	0.874	1.745	0.230

MMP9	185	182	1.200	0.863	1.668	0.278
CXCL10	118	249	1.208	0.839	1.739	0.309
PLEK	170	197	1.105	0.794	1.539	0.553
CXCL1	264	103	1.073	0.756	1.523	0.693
Other hub genes in WGCNA network						
RGS1	249	118	1.806	1.297	2.514	<0.001
CLEC7A	92	275	1.599	1.052	2.429	0.027
LILRB4	118	249	1.522	1.044	2.218	0.028
HCK	99	268	1.491	0.997	2.228	0.049
SLCO2B1	209	158	1.353	0.977	1.875	0.068
C1QC	128	239	1.364	0.950	1.958	0.091
GBP1	113	254	1.378	0.938	2.026	0.101
LAIR1	98	269	1.385	0.931	2.061	0.106
HAVCR2	102	265	1.371	0.930	2.022	0.109
FPR3	235	132	1.294	0.929	1.803	0.127
TNFSF13B	104	263	1.333	0.911	1.950	0.138
TREM2	156	211	1.279	0.912	1.793	0.153
PLA2G7	114	253	1.303	0.903	1.881	0.156
SIGLEC7	253	114	1.276	0.909	1.790	0.158
TM6SF1	234	133	1.265	0.909	1.761	0.162
IFI16	236	131	1.256	0.900	1.751	0.179
MS4A6A	229	138	1.251	0.898	1.741	0.184
CD53	264	103	1.264	0.892	1.790	0.187
MSR1	124	243	1.259	0.879	1.803	0.208
SAMSN1	133	234	1.235	0.864	1.766	0.245
LCP1	191	176	1.192	0.860	1.652	0.290
GPR65	260	107	1.205	0.850	1.706	0.294
SLA	151	216	1.158	0.823	1.630	0.400
IL2RA	130	237	1.158	0.813	1.651	0.415

Table S9 Correlation between key genes and immune factors in TCGA-STAD

Immune factors	*TYROBP*		*C1QB*	
	R	*P*	R	*P*
B cells naive	-0.27	<0.001	-0.24	<0.001
B cells memory	-0.18	0.005	-0.22	0.001
Plasma cells	-0.19	0.004	-0.15	0.026
T cells CD8	0.21	<0.001	0.26	<0.001
T cells CD4 naive	-0.10	0.121	-0.10	0.114
T cells CD4 memory resting	-0.27	<0.001	-0.25	<0.001
T cells CD4 memory activated	0.26	<0.001	0.37	<0.001
T cells follicular helper	0.01	0.887	0.11	0.057
T cells regulatory	0.01	0.758	-0.09	0.146
T cells gamma delta	0.15	<0.001	0.22	<0.001
NK cells resting	0.01	0.044	0.01	0.054
NK cells activated	-0.04	0.862	-0.05	0.690
Monocyte	0.06	0.364	-0.02	0.770
Macrophages M0	0.00	0.945	-0.05	0.421
Macrophages M1	0.23	<0.001	0.36	<0.001
Macrophages M2	0.46	<0.001	0.47	<0.001
Dendritic cells resting	0.20	0.001	0.23	<0.001
Dendritic cells activated	-0.22	0.025	-0.29	0.001
Mast cells resting	-0.01	0.843	-0.01	0.886
Mast cells activated	-0.10	0.144	-0.16	0.014
Eosinophils	0.15	<0.001	0.11	<0.001
Neutrophils	0.14	0.004	0.03	0.175
GZMA	-0.25	<0.001	-0.24	<0.001
PRF1	-0.33	<0.001	-0.32	<0.001
Cytolytic activity	-0.30	<0.001	-0.28	<0.001
CD274	0.49	<0.001	0.61	<0.001
PDCD1	0.55	<0.001	0.65	<0.001
PDCD1LG2	0.72	<0.001	0.80	<0.001
LAG3	0.59	<0.001	0.70	<0.001

Table S10 Univariable and multivariable Cox regression for *TYROBP*, *C1QB* and macrophages

Model	Variables	HR	95%CI		*P*
			Bottom	top	
Univariable Cox model	*TYROBP*	1.455	1.038	2.041	0.029
	C1QB	1.474	1.037	2.097	0.030
	macrophages M1	1.182	0.817	1.712	0.374
	macrophages M2	1.494	1.055	2.116	0.024
Multivariable Cox model 1	*TYROBP*	1.518	1.060	2.174	0.023
	macrophages M1	1.054	0.719	1.545	0.786
Multivariable Cox model 2	*C1QB*	1.550	1.059	2.268	0.024
	macrophages M1	1.033	0.702	1.520	0.869
Multivariable Cox model 3	*TYROBP*	1.399	0.970	2.018	0.073
	macrophages M2	1.340	0.929	1.933	0.118
Multivariable Cox model 4	*C1QB*	1.428	0.976	2.088	0.067
	macrophages M2	1.349	0.939	1.94	0.106
