# Supplementary figures and images for: Identification of TYROBP and C1QB as Two Novel Key Genes With Prognostic Value in Gastric Cancer by Network Analysis
Source: Front Oncol. 2020 Sep 11;10:1765. doi: 10.3389/fonc.2020.01765 (PMC7516284; doi:10.3389/fonc.2020.01765)

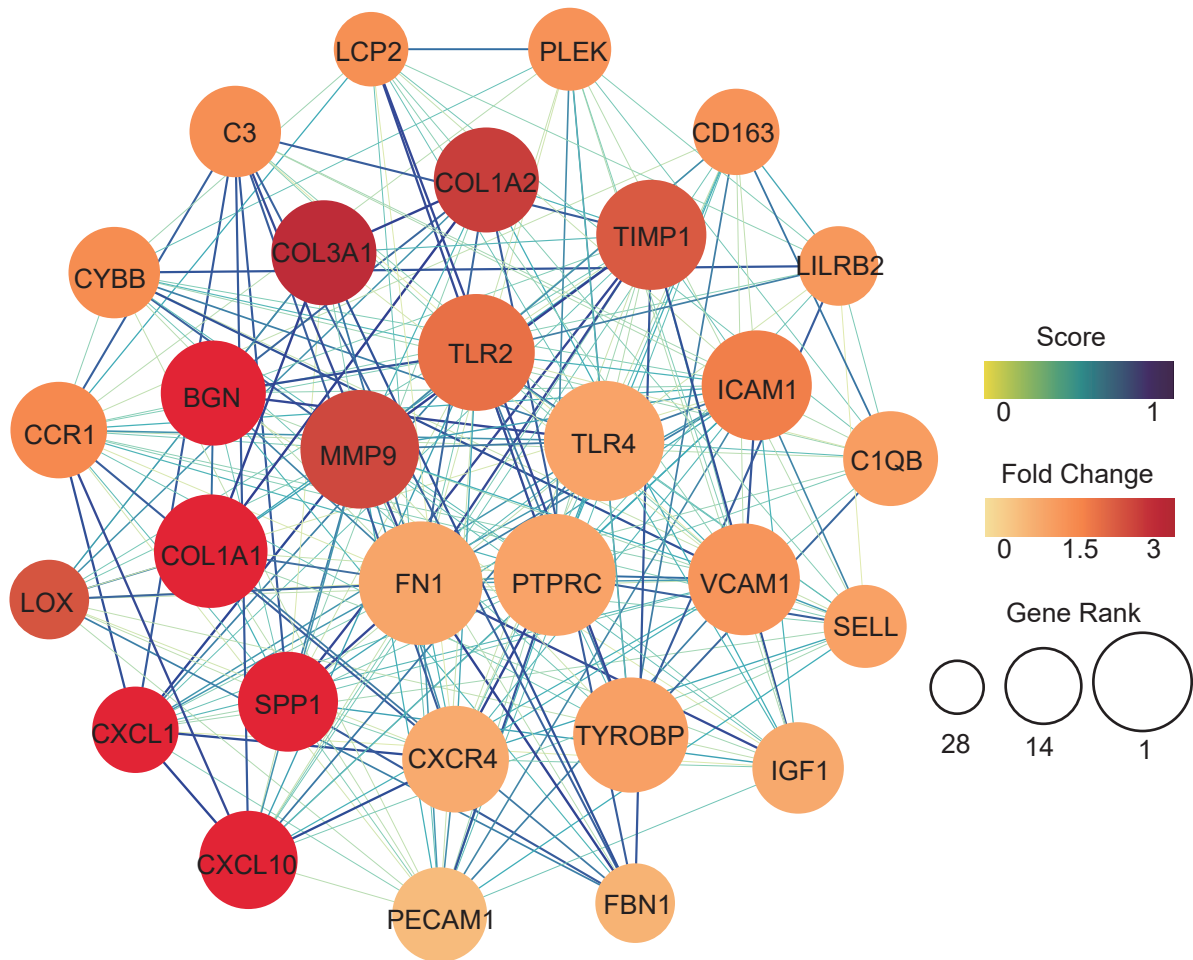

**Supplementary Figure 1** | Identification of top 30 hub genes in PPI network by Degree method.

Supplement: Supplementary file 2 [file Image_1.pdf]

**A**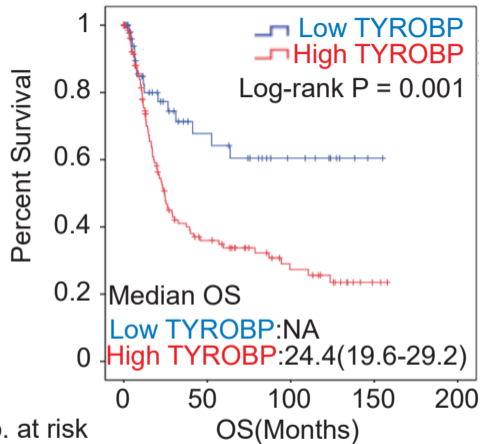**B**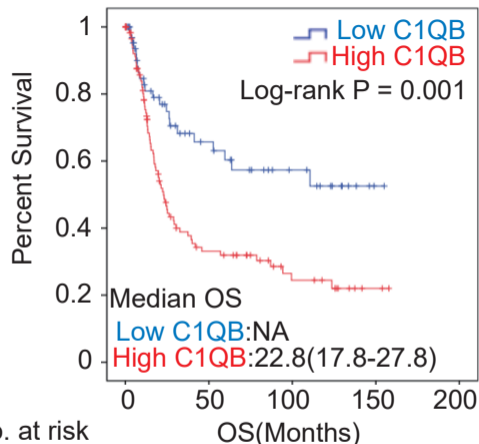

**Supplementary Figure 6** | Survival analysis of key genes in GSE15459. (A) TYROBP. (B) C1QB.

Supplement: Supplementary file 7 [file Image_6.pdf]
