# Supplementary material for: Identification of TYROBP and C1QB as Two Novel Key Genes With Prognostic Value in Gastric Cancer by Network Analysis
Source: Front Oncol. 2020 Sep 11;10:1765. doi: 10.3389/fonc.2020.01765 (PMC7516284; doi:10.3389/fonc.2020.01765)
Supplement: Supplementary file 4 [file Image_3.pdf]

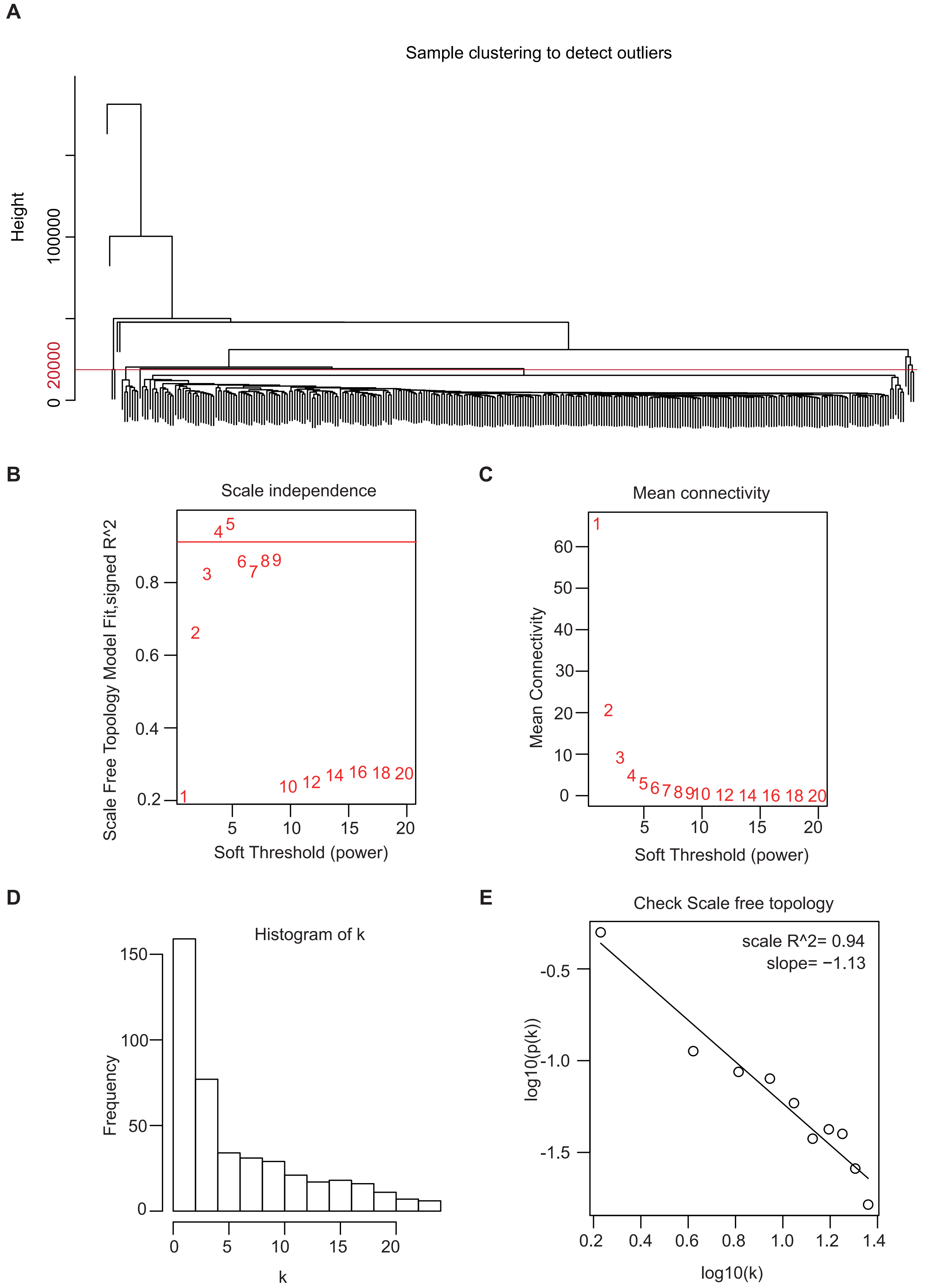

Supplementary Figure 3 | Clustering dendrogram of samples and determination of soft-thresholding power. **(A)** Clustering is based on the common DEGs expression data of TCGA-STAD. **(B)** Analysis of the scale-free fit index for various soft-thresholding powers. **(C)** Analysis of the mean connectivity for various β values. **(D)** Histogram of connectivity distribution when β = 4. **(E)** Checking the scale free topology when β = 4.
