# Supplementary material for: Identification of TYROBP and C1QB as Two Novel Key Genes With Prognostic Value in Gastric Cancer by Network Analysis
Source: Front Oncol. 2020 Sep 11;10:1765. doi: 10.3389/fonc.2020.01765 (PMC7516284; doi:10.3389/fonc.2020.01765)
Supplement: Supplementary file 6 [file Image_5.pdf]

A

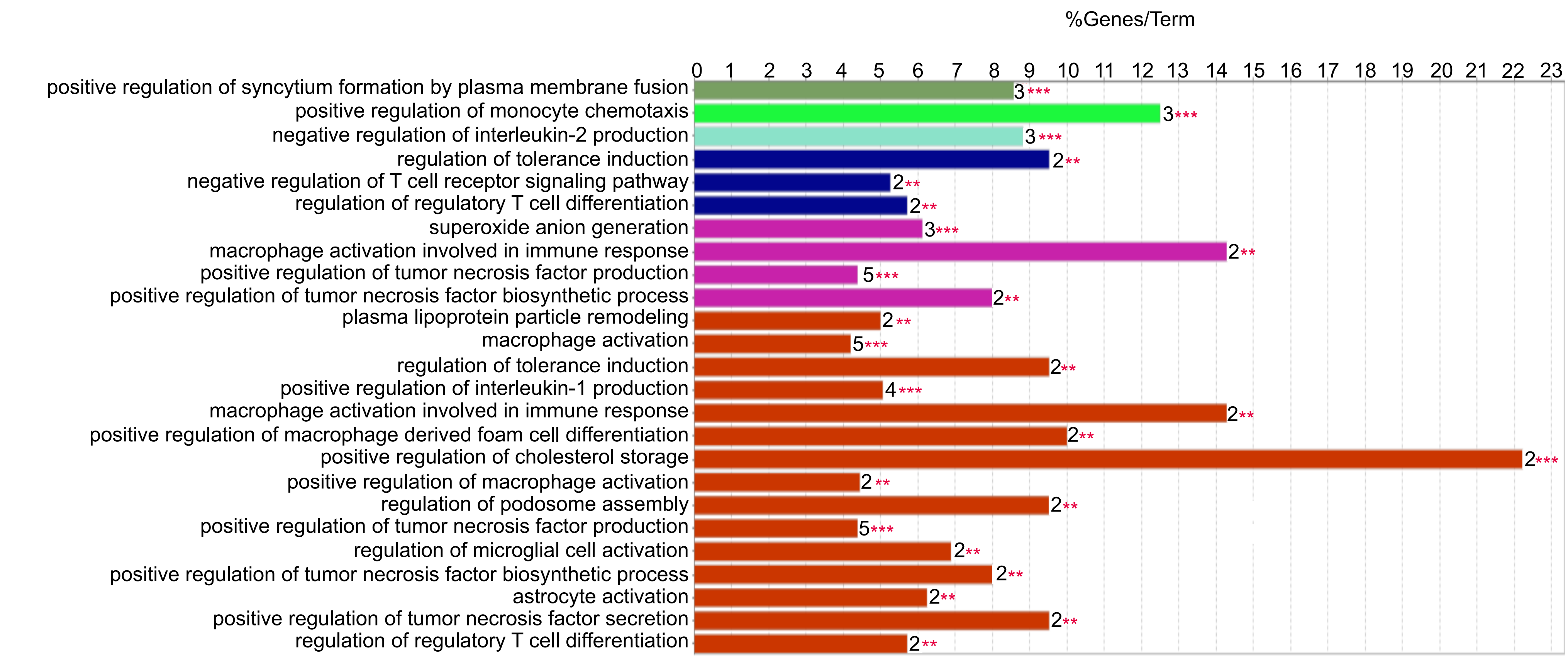

B

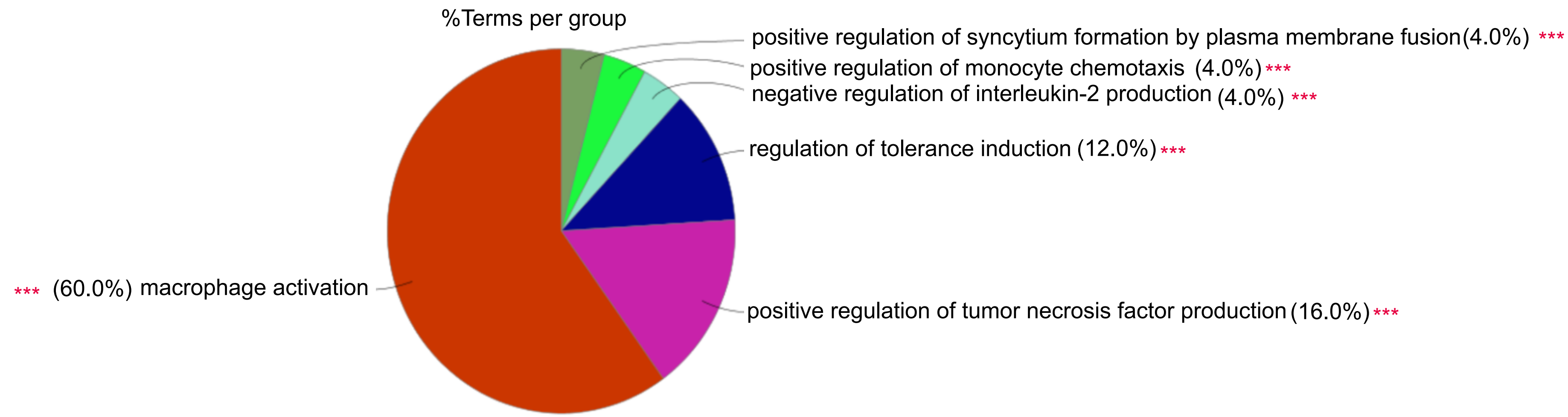

Supplementary Figure 5 | Numbers of genes and terms enriched in the identified pathways of hub genes from WGCNA network. **(A)** Numbers of genes enriched in the identified pathways. **(B)** Numbers of terms enriched in the identified pathways.
