# Supplementary material for: Identification of TYROBP and C1QB as Two Novel Key Genes With Prognostic Value in Gastric Cancer by Network Analysis
Source: Front Oncol. 2020 Sep 11;10:1765. doi: 10.3389/fonc.2020.01765 (PMC7516284; doi:10.3389/fonc.2020.01765)
Supplement: Supplementary file 8 [file Image_7.pdf]

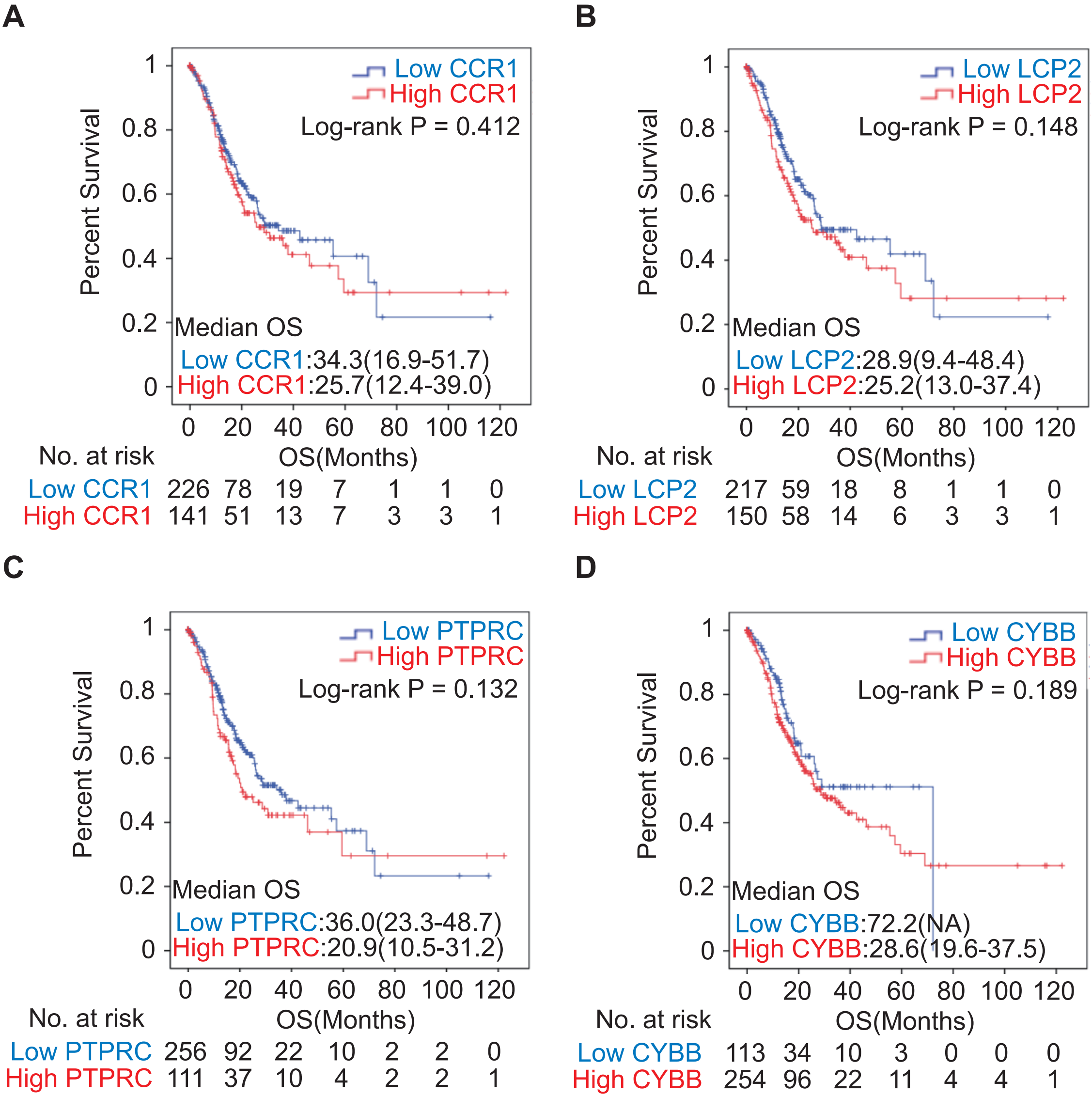

Supplementary Figure 7 | Survival analysis of 4 common hub genes including CCR1, LCP2, PTPRC, CYBB in TCGA-STAD. **(A)** CCR1. **(B)** LCP2. **(C)** PTPRC. **(D)** CYBB.
