# Supplementary material for: Identification of TYROBP and C1QB as Two Novel Key Genes With Prognostic Value in Gastric Cancer by Network Analysis
Source: Front Oncol. 2020 Sep 11;10:1765. doi: 10.3389/fonc.2020.01765 (PMC7516284; doi:10.3389/fonc.2020.01765)
Supplement: Supplementary file 9 [file Image_8.pdf]

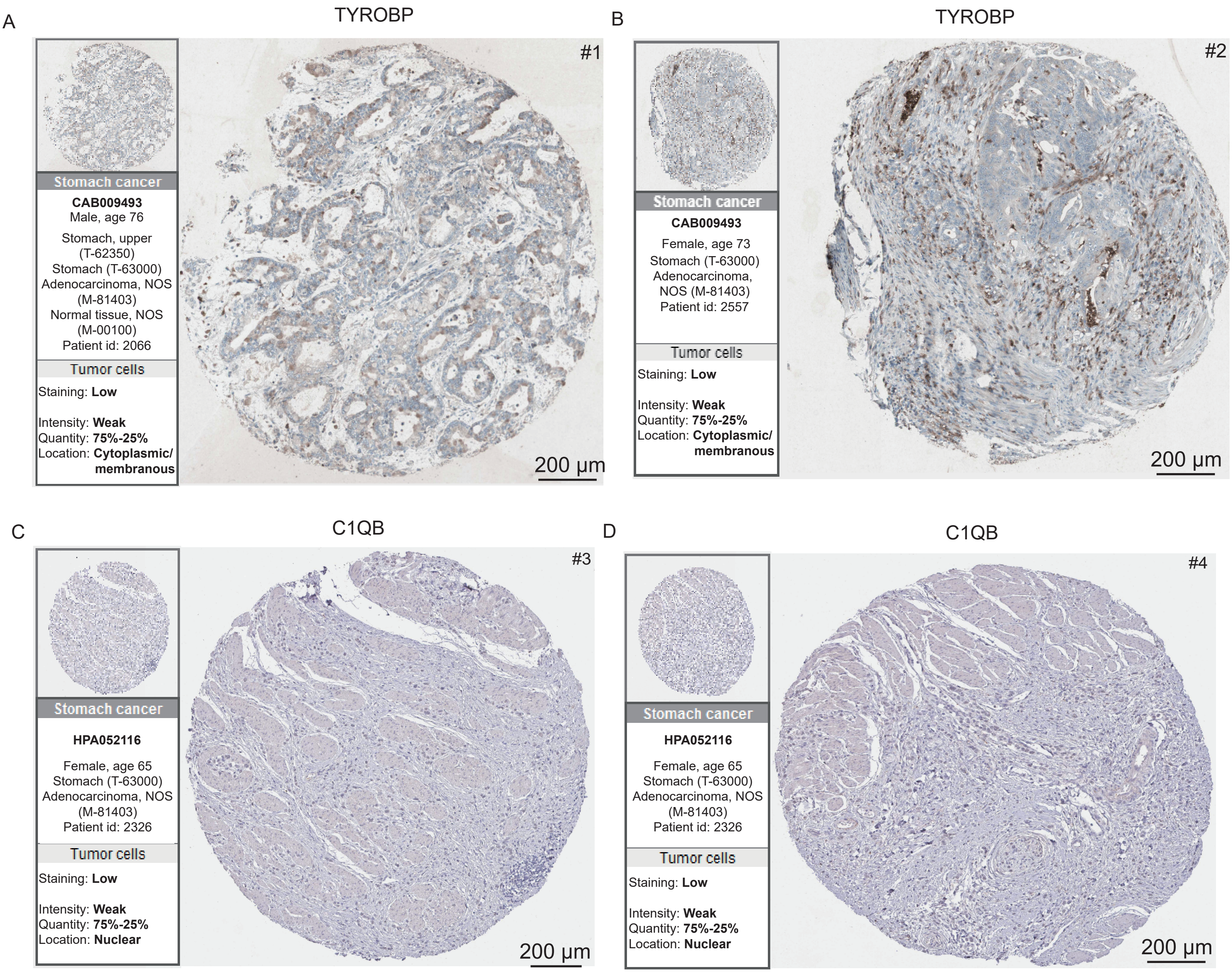

Supplementary Figure 8 | Expression of TYROBP and C1QB in immunohistochemistry from Human Protein Htlas database. (A) TYROBP in #1 slice; (B) TYROBP in #2 slice; (C) C1QB in #3 slice; (D) C1QB in #4 slice.
