# Supplementary material for: Identification of TYROBP and C1QB as Two Novel Key Genes With Prognostic Value in Gastric Cancer by Network Analysis
Source: Front Oncol. 2020 Sep 11;10:1765. doi: 10.3389/fonc.2020.01765 (PMC7516284; doi:10.3389/fonc.2020.01765)
Supplement: Supplementary file 10 [file Image_9.pdf]

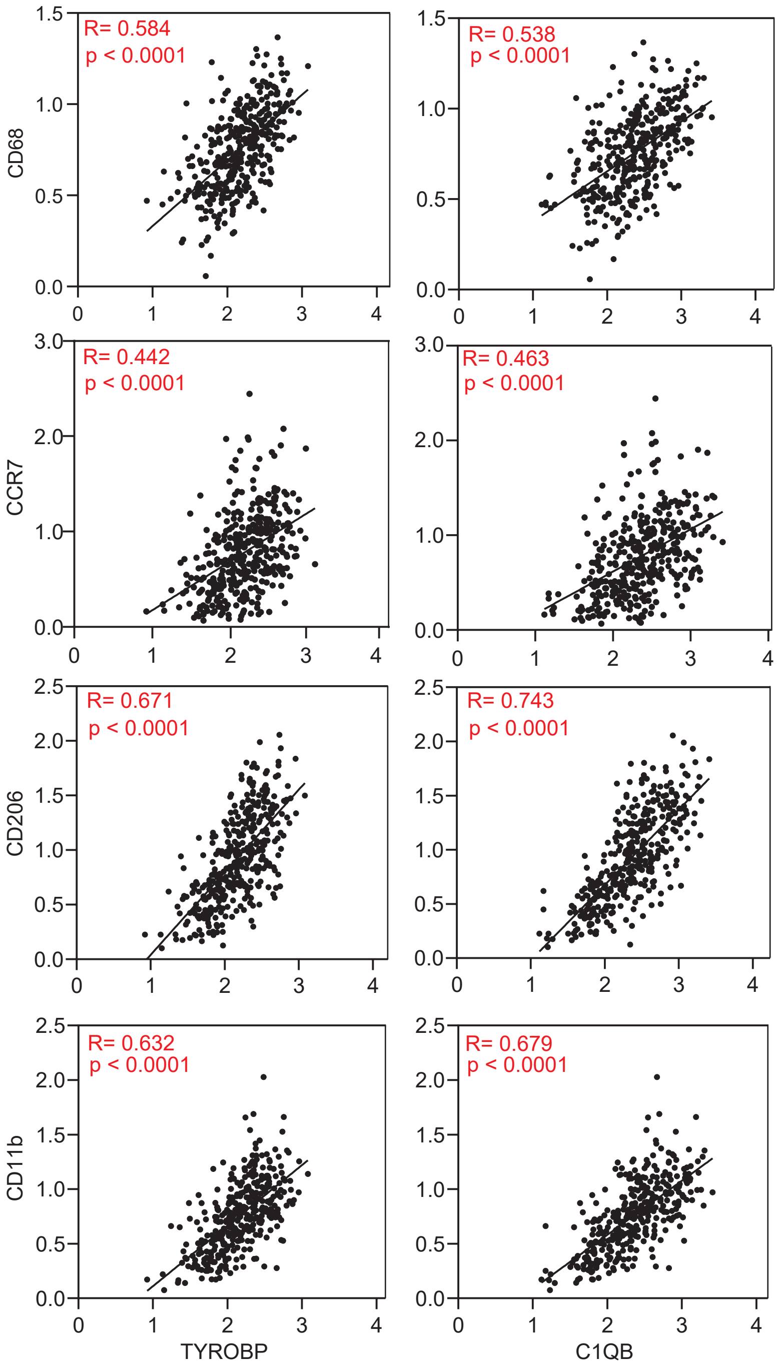

Supplementary Figure 9 | Correlation analysis of TYROBP and C1QB with differential markers of macrophage. CD68, a marker of M0; CCR7, a marker of M1; CD206 and CD11b, markers of M2.
