# Supplementary material for: Identification of TYROBP and C1QB as Two Novel Key Genes With Prognostic Value in Gastric Cancer by Network Analysis
Source: Front Oncol. 2020 Sep 11;10:1765. doi: 10.3389/fonc.2020.01765 (PMC7516284; doi:10.3389/fonc.2020.01765)
Supplement: Supplementary file 11 [file Image_10.pdf]

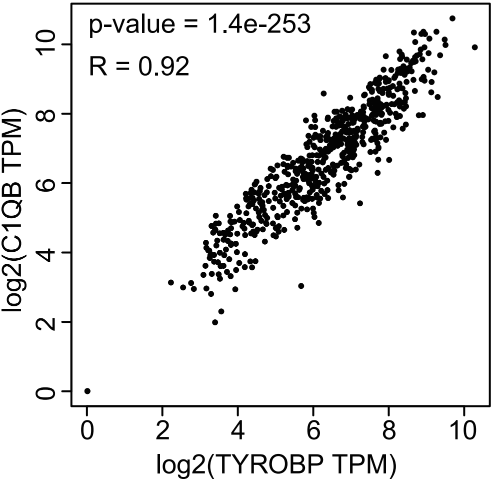

Supplementary Figure 10 | Correlation analysis between the expression of TYROBP and C1QB in GEPIA database. R, spearman's correlation coefficient; TPM, transcripts per million.
