# Supplementary material for: Identification of TYROBP and C1QB as Two Novel Key Genes With Prognostic Value in Gastric Cancer by Network Analysis
Source: Front Oncol. 2020 Sep 11;10:1765. doi: 10.3389/fonc.2020.01765 (PMC7516284; doi:10.3389/fonc.2020.01765)
Supplement: Supplementary file 5 [file Image_4.pdf]

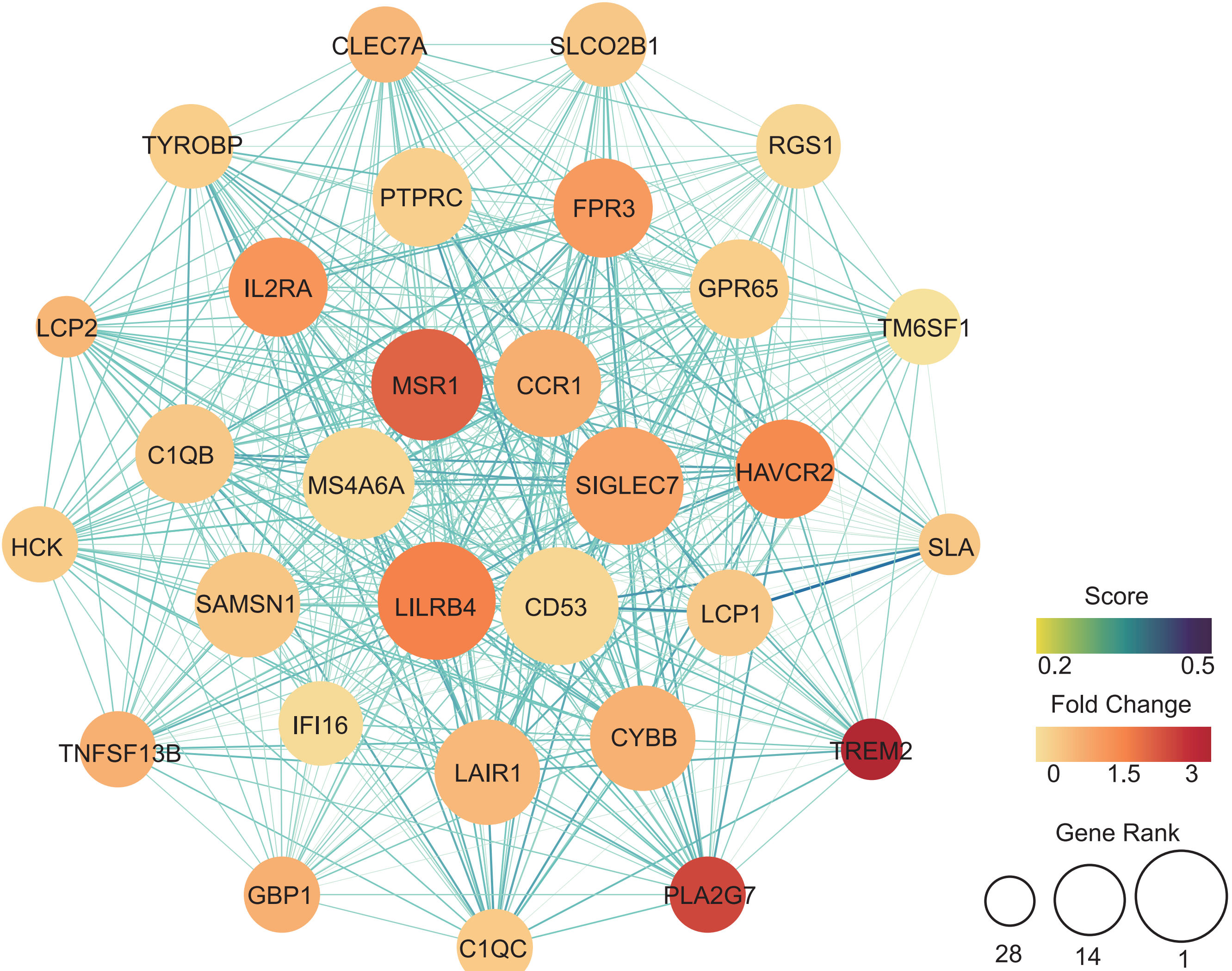

Supplementary Figure 4 | Identification of top 30 hub genes in WGCNA network by Degree method.
